# Evaluation of Aggregated Ag85B Antigen for Its Biophysical Properties, Immunogenicity, and Vaccination Potential in a Murine Model of Tuberculosis Infection

**DOI:** 10.3389/fimmu.2017.01608

**Published:** 2017-11-27

**Authors:** Faraz Ahmad, Swaleha Zubair, Pushpa Gupta, Umesh Datta Gupta, Rakesh Patel, Mohammad Owais

**Affiliations:** ^1^Molecular Immunology Laboratory, Interdisciplinary Biotechnology Unit, Aligarh Muslim University, Aligarh, Uttar Pradesh, India; ^2^Department of Computer Science, Aligarh Muslim University, Aligarh, Uttar Pradesh, India; ^3^BSL-3 Experimental Animal Facility, Department of Animal Experimentation, National JALMA Institute for Leprosy and Other Mycobacterial Diseases (ICMR), Agra, India; ^4^Department of Immunology, National JALMA Institute for Leprosy and Other Mycobacterial Diseases (ICMR), Agra, India

**Keywords:** functional amyloids, immunogenicity, Th1 cytokines, tuberculosis, vaccine development

## Abstract

Protein aggregates have been reported to act as a reservoir that can release biologically active, native form of precursor protein. Keeping this fact into consideration, it is tempting to exploit protein aggregate-based antigen delivery system as a functional vaccine to expand desirable immunological response in the host. Herein, we explored the capacity of aggregated Ag85B of *Mycobacterium tuberculosis* (*Mtb*) to act as a prophylactic vaccine system that releases the precursor antigen in slow and sustained manner. Being particulate system with exposed hydrophobic residues, aggregated Ag85B is likely to be avidly taken up by both phagocytosis as well as fusion with plasma membrane of antigen presenting cells, leading to its direct delivery to their cytosol. Its unique ability to access cytosol of target cells is further evident from the fact that immunization with aggregated Ag85B led to the induction of Th1-dominant immune response along with upregulated expression of qualitatively superior polyfunctional T cells in the mice. Antibodies generated following immunization with aggregated antigen recognized both native and monomeric Ag85B released from protein aggregate. The implicated immunization strategy offers protection at par to that of established BCG vaccine with desirable central and effector memory responses against subsequent *Mtb* aerosol challenge. The study highlights the potential of aggregated Ag85B as promising antigen delivery system and paves the way to design better prophylactic regimes against various intracellular pathogens including *Mtb*.

## Introduction

Tuberculosis (TB) is a major global health problem affecting over one-third population of the world. In general, around 10–15% of the subjects exposed to *Mycobacterium tuberculosis* (*Mtb*) develop active disease, while remaining may act as a carrier of latent form of the infection (1). The human subjects who are on immuno-suppression therapy (*cf*., post organ transplantation) or having co-infection with HIV and other immune-associated complications are more susceptible to full blown TB infection. The emergence of MDR/XDR isolates against available anti-TB antibiotics has further complicated the situation. Bacillus Calmette–Guerin (BCG), the only approved vaccine against TB offers promising prophylaxis against childhood miliary TB, however, remain capricious in providing protection to immunized adults. Despite the admittance of at least 13 potential vaccine candidates in various developmental stages of clinical trials to replace BCG, TB vaccine discovery program is still bleak and necessitates conceptual rejuvenation (2).

Protein aggregates (amyloids) have been implicated in several related human ailments; however, quite a few recent reports have unfolded their functionally beneficial aspects (3–6). Considering that synthesis of amyloidal bodies occurs in a reversible manner, the aggregated or amyloid form of proteins holds enormous potential in the development of future biomedical interventions. For example, partial amyloid form of insulin had been shown to act as a depot for its sustained release for up to 120–140 days in diabetic rats (4). Earlier, Maji et al. (3) had highlighted the utility of amyloid as a depot of gonadotropin releasing hormone. These studies are testament of the fact that amyloidal protein aggregates have potential to release biologically functional monomeric precursor protein. Interestingly, bacterial amyloids (*cf*., curli fibrils) have recently been shown to induce pro-inflammatory IL-17, IL-22, and IL-1β cytokines *via* activation of both TLR2/TLR1 complex and NLRP3 inflammasome (7, 8). More recently, formation of functional amyloid fibrils of adapter protein Imd has been shown to positively regulate the NF-κB-mediated innate immune response in *Drosophila* (6). Reckoning with the fact, we too recently reported that immunization with *in vitro* aggregated form of protein induces production of antibodies in the host that have potential to interact with both; fibril released as well as purified, native protein (5). In addition, we also established that the immunization with aggregated/amyloid form of ovalbumin led to the enhanced antigen-specific proliferation of CTLs and Th1 type of cytokines in BALB/c as well as OVA-TCR transgenic mice (OT-I and OT-II) (5).

Encouraged with its immune-potentiating attributes and also an ability to release precursor protein over extended time period, we sought to evaluate the potential of *in vitro* aggregated form of Ag85B to provide protection against chronic pulmonary TB. Ag85B (or Rv1886c), a major secreted immunodominant protein of *Mtb*, has been extensively probed as a prospective vaccine candidate (9–12). Immunization with rBCG over expressing Ag85B followed by boosting with rAg85B had been reported to offer remarkable protection when compared to that with classical BCG in mice infected with *M. leprae* (10). Besides being classified as a T cell antigen that trigger MHC class 1 pathway (13, 14), the promiscuous nature of Ag85B is likely to complement simultaneous activation of specific CTLs in conjunction with desirable helper T cell activation. In fact, the amyloids/protein aggregates had been reported to possess unique motifs that may interact with pattern recognition receptors, in particular toll-like receptors (TLRs) of APCs, and plausibly regulate subsequent T-cell activation (15).

In the present study, we evaluated immunological outcome of vaccination with aggregated Ag85B antigen in BALB/c mice. The implicated immunization schedule induced robust and long lasting immune response against *Mtb* aerosol challenge in vaccinated animals.

## Materials and Methods

### Reagents

All standard reagents used in the study were purchased from Sigma Aldrich (St. Louis, MO, USA), unless otherwise mentioned. The bacterial culture reagents, Middlebrook 7H9 broth; Middlebrook 7H11 agar, and Oleic Acid Albumin Dextrose and Catalase (OADC) supplement were procured from Difco Laboratories (Sparks, MD, USA). Plastic-wares were purchased from BD Biosciences (San Jose, CA, USA).

### *Mycobacterium bovis* BCG and *Mtb* H_37_Rv Strains

Bacillus Calmette–Guerin (Danish) and *Mtb* H_37_Rv strains were kindly provided by Director, National JALMA Institute for Leprosy and Other Mycobacterial Diseases (NJIL and OMD), Agra, India. *Mtb* was cultured in Middlebrook 7H9 broth containing 0.2% glycerol and 0.05% Tween-80 supplemented with 10% OADC at 37 °C. The H_37_Rv used in the study was passaged through mice on regular basis to ascertain its virulence. Bacterial viability was determined by culturing them on Middlebrook 7H11 medium supplemented with OADC and counting the number of colony-forming units (CFUs).

### Antigens Employed in *Ex Vivo* Stimulation

Whole cell antigens (10 µg/ml) of *Mtb* H_37_Rv and *M. bovis* BCG were used for the stimulation of *ex vivo* cultures of splenocytes from Placebo and BCG immunized control groups, respectively. A known amount (5 µg/ml) of purified recombinant Ag85B [or equivalent amount of Ag85B aggregates (F2)] was used to stimulate the cultures belonging to various experimental immunized groups, respectively, in various cell culture experiments. Whole cell lysates of both *Mtb* and *M. bovis* BCG were prepared by sonication employing Bio-Safety Level-3 (BSL-3) Facility of NJIL and OMD (Agra, India). Purified recombinant Ag85B was procured through BEI Resources (Manassas, VA, USA) under TB research material procurement contract between NIAID (USA) and AMU (India). As per manufacturer, the recombinant protein was expressed in *Escherichia coli* and purified using standard chromatographic techniques followed by endotoxin removal procedures.

### Antibodies and Cytokine Estimation Kits

The following fluorochrome-labeled anti-mouse antibodies were procured from e-Biosciences and BD Biosciences: anti CD3 (145-2C11), anti CD11c (HL3), anti CD4 (GK 1.5), anti CD8 (53-6.7), anti CD44 (IM7), anti CD62L (MEL-14), anti CD80 (B7-1), anti CD86 (GL1), anti F4/80 (T45-2342), anti TNF-α (MP6-XT22), and anti IFN-γ (XMG1.2). Mouse IgG isotyping kit (Cat. No. 550487) and IL-4, IFN-γ, IL-12 BD OptEIA cytokine ELISA kits were procured from BD Biosciences (USA). Monoclonal anti Ag85 complex (CS-90) antibody was procured from BEI Resources (Manassas, VA, USA). Alexa Fluor 488 tagged secondary anti-mouse antibody (Cat. No. A32723) was purchased from Thermo Fisher Scientific (IL, USA).

### Preparation of Fibrillar Aggregates of *Mtb* Ag85B

Fibrillization of Ag85B was executed following the method described previously with slight modifications as standardized in our lab (5). In brief, antigen Ag85B was dissolved and incubated in eppendorf tubes at concentration of 1 mg/ml in PB (10 mM, pH 7.2) for various incubation periods. The tubes containing samples, with their caps sealed with parafilm, were allowed to agitate uninterruptedly at 90 rpm using a laboratory grade orbital shaker (Major Science, Saratoga, CA, USA). All the operations were executed at 25 ± 2 °C. The samples were withdrawn at stipulated time periods. The suspension sample was centrifuged at 15,000*g* for 15 min to obtain the aggregates as pellet. The obtained aggregates were thoroughly washed twice with PB (10 mM, pH 7.2). The formation of fibrillar aggregates was established by employing various spectrophotometric and spectrofluorometric assays. The *in vitro* formed fibrillar aggregates were dissolved in PB (10 mM, pH 7.2) to perform relevant biophysical and immunological studies. All involved procedures were carried out aseptically so as to minimize the possibility of the introduction of toxins or other environmental contaminants.

### Biophysical Characterization of As-Synthesized Ag85B Aggregates

The formation of fibrillar Ag85B was established employing various biophysical methods. Congo Red (CR) binding assay was performed with slight alteration to original protocol (16) to confirm the amyloidal nature of aggregated Ag85B antigen. Briefly, an aliquot of free antigen or synthesized amyloidal aggregates; pre-incubated for 30 min with CR solution (50 µM), was scanned between 200 and 700 nm on double beam UV-Vis spectrophotometer (Model Lambda 25, Perkin Elmer, USA). Absorbance obtained for CR alone was subtracted from absorbance recorded for test protein samples.

The formation of polymeric amyloidal/aggregated assembly was further established by Thioflavin-T (ThT) fluorescence assay according to published procedure (16). An aliquot representing formed amyloidal assembly was incubated with ThT dye (50 µM) for 30 min. The samples were excited at 450 nm, and their emissions were monitored in range of 460–560 nm using a spectro-fluorometer (Model RF 5405-PC, Shimadzu, Japan).

Measurement of Rayleigh Scattering or Fluorescence Intensity (FI) at 350 nm has been generally employed to follow protein aggregation (17). Rayleigh Scattering measurements were performed on F-4500 fluorescence spectrometer (Hitachi, Japan) at room temperature (RT) using a cell with a 1-cm light path. Turbidity measurements were also performed; the incubated samples collected at various time points were monitored by taking UV absorbance at 350 nm using a UV-Vis spectrophotometer (Lambda 25, Perkin Elmer, USA) in a 1-cm path length cuvette at 25 °C.

### *In Vitro* Release Kinetics of As-Synthesized Ag85B Aggregates

Native *Mtb* Ag85B was dissolved in PB (pH 7.2) at 1 mg/ml concentration in an eppendorf tube and incubated at RT with constant shaking. Samples from the fibrillation reaction were withdrawn at various time points for analysis. The fibrillar aggregates obtained by centrifugation at 15,000*g* were washed twice with PB (pH 7.2) and subsequently dissolved in 1 ml of the same buffer and finally the tubes were sealed with parafilm and kept at RT in static condition. The kinetics of antigen released from the fibrillar pellet into surrounding PB (pH 7.2) was monitored spectrophotometrically at 280 nm (4). The supernatant obtained after centrifugation was analyzed at various time points for a period of 15 days using UV/VIS spectrophotometer (Model Lambda 25, Perkin Elmer, USA).

### Dot Blot Analysis

Both, purified native as well as aggregate/fibril released (from 24 h as-synthesized fibril) forms of Ag85B antigens were loaded onto PVDF strips and subsequently allowed to dry at RT. The strips were rinsed briefly in phosphate-buffered saline (PBS; pH 7.4) containing Tween-20 (PBST) and incubated overnight at 4 °C in 5% non-fat dry milk in PBST to block the residual binding sites on the membrane. The strips were again rinsed three times in PBST. The strips coated with both forms of Ag85B antigen were probed with Ag85B specific polyclonal antibodies purified from the sera of Ag85B fibril (F2) immunized animals. After stipulated incubation, the strips were washed thrice in PBST and further incubated for 1 h at 37 °C with horseradish peroxidase-tagged goat anti-mouse antibody (1:5,000). The strips were washed with PBST three times and finally developed on X-ray film (Amersham, UK) employing ECL kit (Bio-Rad, USA). A dot blot (black spot) at the site where the antigen was spotted was considered as positive result. A non-specific protein, bovine serum albumin (BSA) was also spotted onto membranes as negative control in all dot blot assays.

### *In Vitro* Macrophage Uptake and Immuno-Localization Studies

Uptake of fibrillar Ag85B by RAW264.7 cells was assessed employing both- flow cytometry and fluorescence microscopy. For flow cytometry, antigens (both F1 and F2) were pre-labeled with fluorescein isothiocyanate (FITC) by mixing 1 equivalent of either antigen with 2 equivalents of FITC in NaHCO_3_ buffer (pH 8.5). The solution was allowed to mix gently at RT for 1 h, and then purified by three times of ultrafiltration using Amicon Ultra-15 centrifugal filter units with 10 kDa cutoff (Millipore) against PB. Labeled antigens (approximately 10 µg each) were subsequently incubated with RAW264.7 cells (1 × 10^5^) for 6 h in a 6-well plate, followed by three washings with PBS and subsequent fixation with 2% paraformaldehyde (PFA). Consequently, cells were washed again with PBS, briefly trypsinized, harvested, and suspended in FACS buffer for acquisition. In unstained control well, cells were incubated with unlabeled native antigen (F1) under similar experimental conditions.

Alternatively, for microscopic study of fibril uptake, cells cultured on cover slips overnight were allowed to interact with fibrillar Ag85B for 6 h at 37 °C, 5% CO_2_. After stipulated incubation, cells were harvested and subsequently incubated in ThT working solution (50 µM) for 30 min in dark at RT. Appropriate controls (native antigen with ThT and ThT alone in buffer) were also included in the assay. Thereafter, cells were fixed with 2% PFA and subsequently washed and mounted with UltraCruz mounting medium containing DAPI (Santa Cruz Biotechnology, USA) for fluorescence imaging.

For immuno-localization experiment, fibrillar Ag85B was incubated with macrophages for 6 h (at 37 °C, 5% CO_2_). Subsequently, RAW264.7 cells were incubated with solution of primary antibodies (monoclonal) against native Ag85B in permeabilization buffer (BD Perm/Wash, San Diego, CA, USA) for 1 h at RT followed by extensive washing with PBS. Next, cells were further incubated for 1 h at RT with Alexa Fluor 488 labeled secondary anti-mouse antibody (Thermo Fisher Scientific, IL, USA). After extensive washing, cover slip was mounted with UltraCruz mounting medium containing DAPI on the glass slide and subsequently imaged with fluorescence microscope (Axio Imager 2, Carl Zeiss, Germany).

### Animals and Immunization Schedule

Total of 70 female, inbred BALB/c mice were obtained from Animal House of NJIL and OMD (ICMR), Agra, India. The animals were segregated and immunized in following four groups (*n* = 17 per group), *viz*., native Ag85B emulsified with Incomplete Freund’s Adjuvant (IFA) herein designated as F1, fibrillar Ag85B (F2), PB administered (Placebo), and BCG (Danish) immunized (once with 1 × 10^6^ cells) group. The animals were immunized subcutaneously at the base of their tail with suspension corresponding to 20 µg of antigen in a volume of 100 µl PB per animal. Three weeks later, the animals were boosted with corresponding formulation of antigen (20 μg/animal) using the same route of administration.

### Ethics Statement

All animal experiments were approved by the Institutional Animal Ethics Committees of Interdisciplinary Biotechnology Unit, AMU, Aligarh, India and National JALMA Institute for Leprosy and Other Mycobacterial Diseases, Agra, India. All animal experiments were performed according to the National Regulatory Guidelines issued by CPCSEA. Our approval ID was 332/CPCSEA, Ministry of Environment and Forest, Govt. of India.

### Establishment of Infection and Determination of Residual Mycobacterial Load in Lungs and Spleen of Immunized Mice

One week post booster, mice representing various experimental and control groups were challenged with virulent *Mtb* H_37_Rv through aerosol route. Suspension corresponding to bacterial count of 5 × 10^7^/ml in a volume of 10 ml normal saline was added to the venture nebulizer unit of the Aerosol Inhalation Exposure System (Glas-Col, USA) to deposit ≈100 bacilli in lungs of each mouse. To confirm the viability and numbers of delivered bacilli in their lungs; two animals were sacrificed before 16 h post infection (Day 1), and their organ homogenates were plated onto 7H11 agar plates. To evaluate the protective efficacy of in-house developed candidate vaccine, bacterial load in lungs and spleen of experimental animals was determined at 10 weeks post challenge. After stipulated time period, four animals from each group were sacrificed; their spleen and lungs were removed aseptically and homogenized in 7H9 media. Four different dilutions of prepared homogenate were plated onto 7H11 agar plates supplemented with OADC. In BCG (Danish) immunized group, thiophene carboxylic acid hydrazide (TCH) at concentration of 2 mg/ml was added to inhibit the growth of BCG. All the plates were incubated for 3–4 weeks at 37 °C in CO_2_ incubator (Galaxy 180S, Eppendorf, Hamburg, Germany) with constant supply of 5% CO_2_. After stipulated incubation period, colonies were counted to calculate the bacterial load. Bacterial loads were interpreted and expressed as means ±log_10_ CFUs/g in lungs and spleen of infected animal.

Representative lung and spleen tissue from each group euthanized at 10 weeks post challenge was also preserved in 10% buffered formalin for histopathological analysis.

### Isolation of Splenic Lymphocytes and APCs

Mice from each experimental group (*n* = 3) were sacrificed at various time points, i.e., 5 days post booster and weeks 2, 6, and 10 post challenge to assess immunological outcome in the immunized animals. Single cell suspension of the spleens was prepared according to published procedure (18). Briefly, spleens from animals representing various groups were macerated using frosted glass slides and passed through 70 µM cell strainer to get single cell suspensions. The cell suspension was treated with ACK lysis buffer for erythrocyte lysis. The cells were washed with Hanks Balanced Salt Solution three times and re-suspended in complete RPMI 1640 medium. Splenic DCs were isolated by magnet assisted negative selection using BD IMag™ Mouse Dendritic Cell Enrichment Set-DM (Cat. No. 557955) following manufacturer’s protocol. The isolated CD11c^+^ DCs were then checked for their co-stimulation potential using flow cytometry.

### Antibody Isotype Determination in Sera of Immunized Animals

Mice were bled at various time intervals (post immunization and post challenge) and their sera were analyzed for the presence of Ag85B-specific antibodies. Subsequently, the antibodies were analyzed for their isotypes using kit purchased from BD Biosciences. Briefly, 96-well micro-titer plates were coated (in duplicate) with isotype specific monoclonal antibodies in carbonate bicarbonate buffer (0.05 M, pH 9.5) and incubated overnight at 4 °C. After washing and blocking steps, the plates were incubated with 100 µl test sera from each animal from each group and positive control (supplied with kit) at RT for 1 h. After excessive washing of the plates, 100 µl of (1:100 dilution of stock) rat anti-mouse IgG antibodies were added in each well and incubated for 1 h at RT. The plates were washed again followed by adding 100 µl of substrate solution (supplied with kit) and were finally incubated for 3–10 min at RT. The reaction was stopped by the addition of 50 µl of stop solution (1M phosphoric acid). On addition of stop solution, the absorbance of as-formed color complex was immediately read at 450 nm with a micro-titer ELISA plate reader (Bio-Rad, USA).

### Lymphocyte Proliferation Assay

Isolated lymphocytes from each group of animals were seeded into 96-well plates in triplicate at the density of 1 × 10^6^ cells/well and cultured with corresponding matching antigen preparation for 68 h. In negative wells, only PBS was added. An aliquot (100 µl) of MTT [3-(4,5-dimethylthiazol-2-yl)-2,5-diphenyltetrazolium bromide, a tetrazole] reagent (from 5 mg/ml stock) was added to each well and incubated for another 4 h at RT. The supernatant was removed, and a 100-µl mixture of DMSO and isopropanol (1:1) was added in each well to obtain homogenized solution. Absorbance was immediately measured thereafter at 570 nm. Cell proliferation was expressed as Stimulation Index (SI) = OD of experimental well/OD of negative well.

### Cytokine Assay: Assessment of Antigen Induced Cytokine Profile

Both Th1 and Th2 cytokines induced in splenocytes culture supernatants (belonging to various experimental groups) upon their co-culture in the presence of the respective recall antigens were estimated using BD OptEIA sandwich ELISA kits (BD Biosciences). Briefly, 100 µl of the purified capture antibodies were adsorbed overnight on polystyrene micro-titer plates (Maxisorp, Thermo Scientific) at 4 °C in carbonate buffer of pH 9.5. Plates were washed five times with PBST and blocked with 1% BSA. After the usual steps of washing, 100 µl of the supernatant (isolated from cultured splenocytes after 48 h) was dispensed in each well. After stipulated incubation time, the plates were thoroughly washed and incubated with biotinylated polyclonal goat anti-mouse detection antibody. Afterward, the plates were washed three times with PBST. Subsequently, 100 µl of streptavidin-HRP conjugate was added to each well and plate was incubated for 30 min at RT. The plates were again washed three times with PBST and finally colored complex was developed with tetra methyl benzidine (TMB). The absorbance was read at 450 nm with a micro-titer ELISA plate reader (Bio-Rad).

### Assessment of Cell Surface Markers on Target Cells Employing Flow Cytometry

The splenocytes were harvested and stained for flow cytometric analysis following protocol provided by BD Biosciences. Briefly, 1 × 10^6^ splenocytes were washed twice with FACS staining buffer (PBS with 1% BSA and 0.1% sodium azide). Cells were incubated with Fc block (2.4G2) or with FITC/PE/PerCP-tagged monoclonal antibodies against (CD3, CD4, CD8, CD44, CD62L, CD80, CD86) for 30 min at 4 °C. After washing, cells were fixed with 4.0% paraformaldehyde (PFA). The flow cytometry data were acquired using either FACS Aria-II or LSR-II (BD Biosciences) platforms and minimum of 20,000 events were recorded for each sample. Data were further analyzed with FACS DIVA (BD) software. The total number of cells of a definite phenotype (CD4^+^CD44^high^CD62L^low/high^, CD8^+^CD44^high^CD62L^low/high^) were calculated by taking the percentage of the gated cell population, as determined by flow cytometry, multiplying them with the total number of cells obtained, and finally dividing the furnished result by the number of events recorded, as described elsewhere (19).

### Intracellular Cytokines Staining

T lymphocyte population from immunized mice were collected, washed with PBS, and stained for surface markers, *viz*., CD3, CD4, and CD8, followed by fixation using BD Cytofix buffer. Thereafter, cells were permeabilized in BD Perm/Wash buffer, followed by staining to probe intracellular IFN-γ and TNF-α in the examined cells. BD GolgiStop solution was added for last 4 h of incubation before collecting the cells for staining and was removed subsequently through washing.

### Histopathological Study

The experimental animals were sacrificed and their lungs were perfuse fixed in 10% buffered formalin. Tissue blocks (of 3 mm × 5 mm dimensions) were processed for paraffin embedding, subsequently; 10-mm thick sections were cut with rotary microtome. Sections were subjected to conventional as well as Ziehl–Neelsen staining to identify and to estimate the relative load of the acid-fast bacilli (AFB). Stained sections were observed under light microscope (Olympus BX-40, Japan). Observations were recorded and interpreted independently by a senior histopathologist in the form of photomicrographs from granuloma positive regions of the samples.

### Statistical Analysis of Data

The data of various immunological studies (pertaining to various immunized groups) was compared employing both two-way or one-way ANOVA (as appropriate) followed by Bonferroni’s multiple comparison test using Graph Pad Prism Software (version 5.0). Data presented from various immunological assays are representative of at least three animals from each group. The *p* values, <0.05(*), <0.01(**), <0.001(***) were considered as significant for analysis and interpretation of experimental data.

## Results

### Biophysical Characterization of *In Vitro* Synthesized Ag85B Aggregates

**Figure 1:**
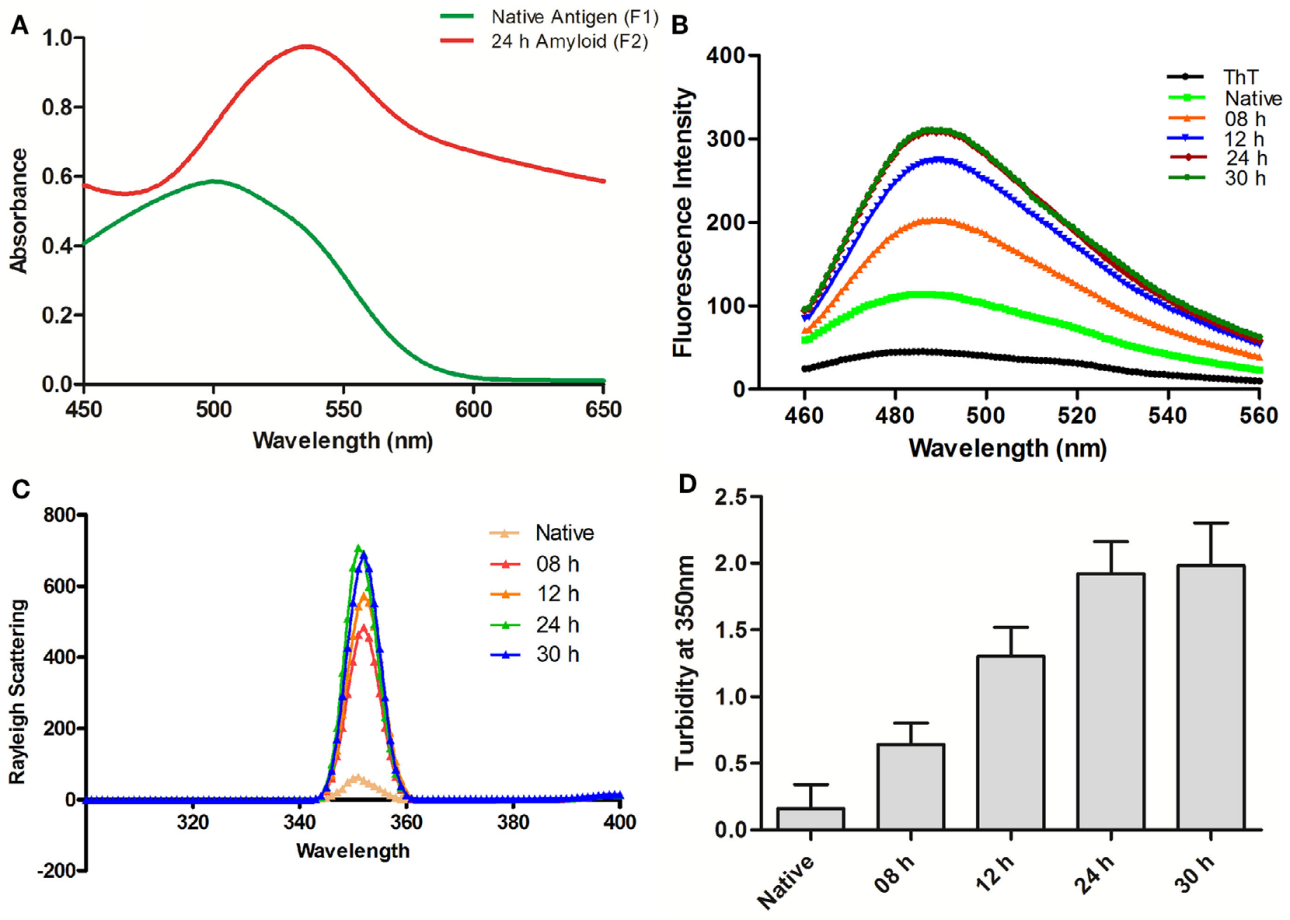
Biophysical characterization of Ag85B aggregates. **(A)** Absorption spectra of Congo Red dye bound to aggregated amyloidal Ag85B. The characteristic spectral “Red Shift” confirms the presence of β-sheet rich amyloidal core. **(B)** Amyloidal aggregate formation as monitored by Thioflavin-T fluorescence spectroscopy. Gradual increase in ThT emission intensities, as obtained by excitation at 450 nm and emission in the range of 460–560 nm, an indication for sequential maturation of Ag85B fibril at various time points. **(C)** Rayleigh Scattering characteristic at 350 nm for Ag85B aggregates. Data pertaining to various samples obtained from simulated reaction process at various time points confirm that the extended agitation gradually induced aggregation of soluble Ag85B. **(D)** Turbidity profile of synthesized aggregates. Various aggregated forms of Ag85B (formed at various incubation stages) were also scanned for increase in the turbidity of protein suspension. Error bars exhibit ±standard errors of means (±SEM). Three independent experiments were performed for each sample and data are representative of at least two independent experiments with similar observations.

Ag85B protein aggregates obtained by continuous agitation for 24 h were evaluated for presence of amyloid specific signature peak shift employing CR binding assay. In general, binding of CR to amyloid results in the characteristic “red shift” change in the spectrum (16). In concordance with the fact, the synthesized fibrillar Ag85B antigen exhibited explicit shift in the absorbance maximum from ≈490 to ≈540 nm as revealed in UV-VIS spectrophotometric analysis (Figure 1A). The characteristic shift in absorbance maxima indicates the presence of β-sheet rich structure in the as-synthesized Ag85B amyloid fibril.

Next, we evaluated Ag85B based fibrillar aggregates for their propensity to bind a benzothiazole based Thioflavin-T (ThT) dye. In concordance with CR assay, ThT binding is considered as a method of choice for the characterization of amyloidal structures (3). ThT, when co-incubated with cross-β-sheet harboring amyloid fibrils, fluoresces strongly with excitation and emission maxima at approximately 440 and 490 nm (16, 20). Specifically, the assay measures the increase in fluorescence intensity of ThT upon binding with amyloid fibrils. Amyloidal protein assembly inherits an intrinsic property to expose hydrophobic residues that foster its interaction with ThT, and exhibit characteristic heightened fluorescence.

In next set of the study, the synthesized fibrillar Ag85B in PB (10 mM, pH 7.2) was excited at 450 nm and the emission was recorded in range of 460–560 nm to validate the formation of amyloidal assemblies. On excitation at 450 nm, significant rise in fluorescence intensity was observed in various aggregated forms of Ag85B over its native form. The observed synchronous increase in fluorescence intensity with incubation time suggests sequential transformation of protein into aggregated amyloidal structure. As observed, the increase in fluorescence intensity got saturated beyond 24 h, suggesting the formation of mature amyloid fibril at 24 h incubation (Figure 1B). Moreover, a rough estimate suggests that ≈85–90% of precursor protein was incorporated into formed fibril, as deduced by analyzing supernatant for protein content employing BCA assay (data not shown).

### Synchronous Increase in Rayleigh Scattering and Turbidity Affirms the Formation of Fibrillar Aggregates of Ag85B

Measurement of Rayleigh Scattering at 350 nm has been widely employed to follow protein aggregation (17). In this context, the extent of light scattering was measured for the Ag85B antigen based aggregate samples, withdrawn at various time intervals from aggregation reaction setup (upto 30 h). With increase in incubation time, an enhancement in fluorescence intensity was observed in corresponding samples. When compared to solution of native antigen, fluorescence intensity was found to increase consistently with incubation time 8 h onward and get saturated after 24 h. As shown in Figure 1C, aggregates collected at various time lapse exhibited more than sevenfold increase in fluorescence intensity in comparison to native Ag85B in a time-dependent manner.

The turbidity measurements determined at 350 nm for Ag85B obtained at various time points during the simulated aggregation reaction revealed a pattern in concordance with fluorescence intensity measurements thus offering further evidence in support of fibrillar aggregate formation (Figure 1D).

### Aggregated Ag85B Offered Depot Effect and Releases Precursor Antigen in Slow and Sustained Manner

**Figure 2:**
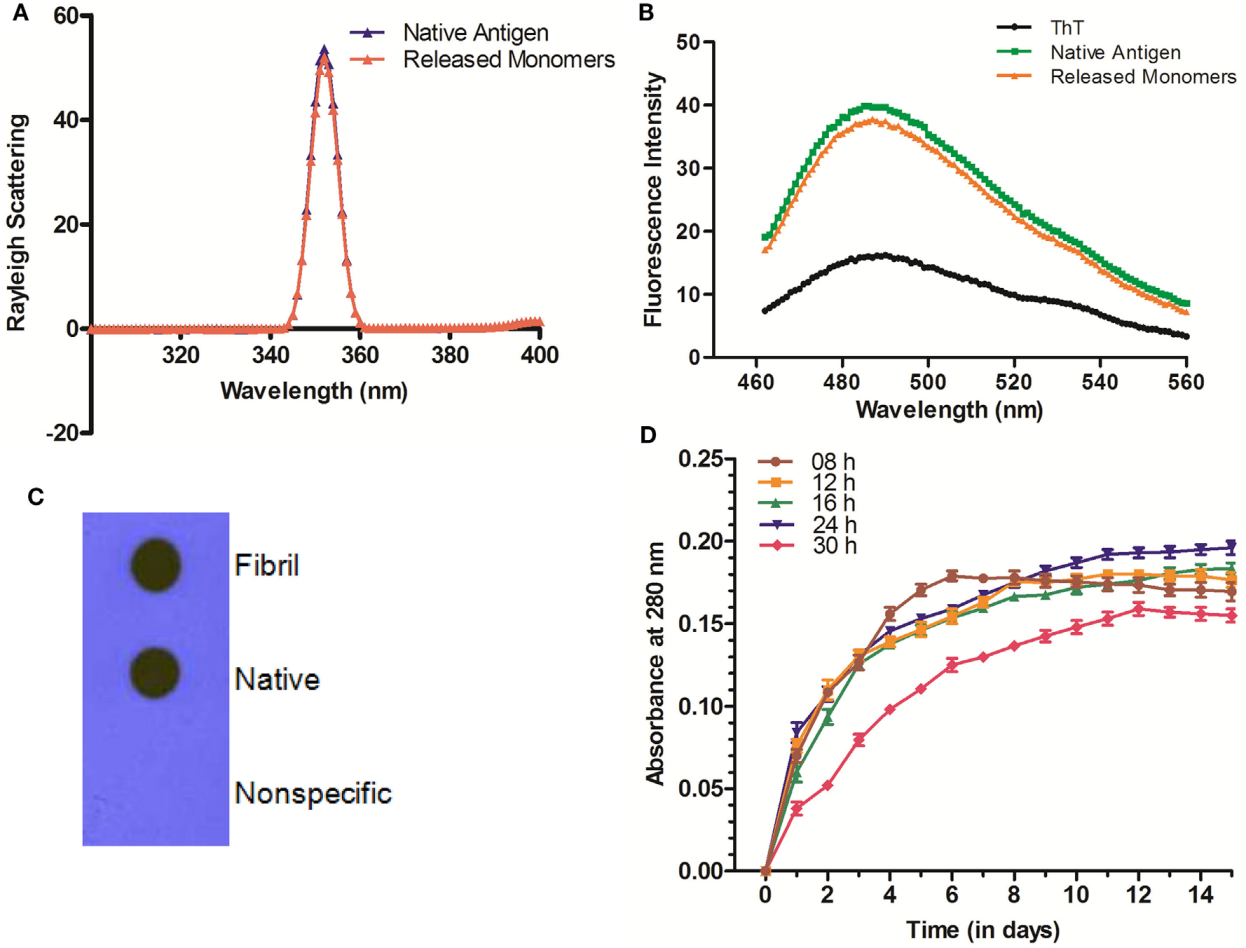
Biophysical properties and release profile of monomeric Ag85B released from fibrillar aggregate. **(A)** Rayleigh Scattering profile of released antigen. Rayleigh Scattering characteristics of monomeric Ag85B, released from aggregated amyloidal core, had biophysical properties overlapping to that of native, free form of antigen. **(B)** ThT binding profile of released monomeric Ag85B. Monomeric Ag85B pooled from *in vitro* release assay was scanned between 460 and 560 nm. The ThT-binding profile of released monomeric Ag85B from fibrillar depot displayed characteristic fluorescence spectra similar to that of the precursor native antigen. The experiment was repeated thrice and same results were obtained each time. **(C)** Dot blot assay to ascertain antibody specificity. Antibodies generated upon immunization with aggregated Ag85B were reacted with both- precursor native as well as released monomeric form of Ag85B from amyloidal depot. A non-specific protein (bovine serum albumin) was coated onto membrane as a control and allowed to react with the same antibody. Data are representative of three independent experiments with similar observations. **(D)** Aggregated Ag85B depot releases monomeric native precursor in the surrounding milieu in a sustained manner. *In vitro* release kinetics of Ag85B from its various time point aggregated forms as well 24 h fibril over a period of 15 days. Three independent experiments were carried out and data are reported as means of two independent experiments with similar observations, where error bars represents ±standard errors of means (±SEM).

The *in vitro* synthesized fibrillar Ag85B formulation was evaluated for its ability to release the native antigen *in vitro*. The release of native Ag85B from its aggregated form (formed at pH 7.2) was monitored *in vitro* spectrophotometrically at 280 nm to assess their sustainability under simulating biological ambience. Among various preparations tested, the fibrillar aggregates of Ag85B protein generated by 24 h shaking incubation released native like protein in a highly sustained manner. Release of native Ag85B from various aggregated forms (8–30 h incubation period), was monitored constantly upto 15 days (Figure 2D). Aggregates obtained at 8 h incubation exhibited burst release for initial 5–6 days that dropped down drastically afterwards. The aggregates formed both at 12 and 16 h time periods released the parent protein steadily with peaks at day 11 for 12 h aggregate and at day 13 for 16 h aggregate, respectively, and attained plateau subsequently. Interestingly, fibrillar aggregates obtained upon 24 h incubation exhibited superior release kinetics and were able to sustain the release of precursor native protein even after 15 days (Figure 2D). The aggregates generated at 18, 20, and 22 h showed closely similar release profile to that of 24 h aggregate (data shown only for 24h aggregate in the figure for sake of simplicity). Importantly, the aggregates obtained beyond 24 h (26, 28, and 30 h aggregate) were found to release native protein monomers feebly as compared to their predecessor (24 h aggregate), as a result the release of monomeric protein was poor and declined in a period of around 10 days (data shown only for 30 h to avoid ambiguity). The observed feeble release pattern exhibited by late stage aggregates (such as 30 h aggregate) implicitly suggests that on attainment of super-maturation state, the higher order fibrillar amyloids fail to release native protein monomers as efficiently as their immediate predecessors.

### The Fibrillar Depot Released Ag85B Exhibits Native Antigen Like Conformation

The released Ag85B from simulated amyloidal core was analyzed for light scattering in solution (10 mM PB, pH 7.2) at 350 nm. In concordance with earlier report (5), the obtained profile resembled to that of native antigen (Figure 2A). This further confirms the notion that synthesized fibrillar Ag85B indeed released native antigen in the surrounding environment. Furthermore, the results from ThT binding studies suggest that the monomers released from fibril depot behave almost in a fashion similar to that of native antigen (Figure 2B). Besides, the nature of the released protein from fibrillar Ag85B *in vitro* was further characterized by dot blot assay. The dot blot investigation suggested that the antibodies generated upon immunization of mice with fibrillar Ag85B recognized both parent (native) as well as released (dislodged from fibril) forms of Ag85B antigen (Figure 2C). The dot blot analysis further support the claim that the antigen released from Ag85B fibrillar depot was structurally identical to the native precursor.

### Interaction of Fibrillar Antigen with RAW264.7 Macrophages

**Figure 3:**
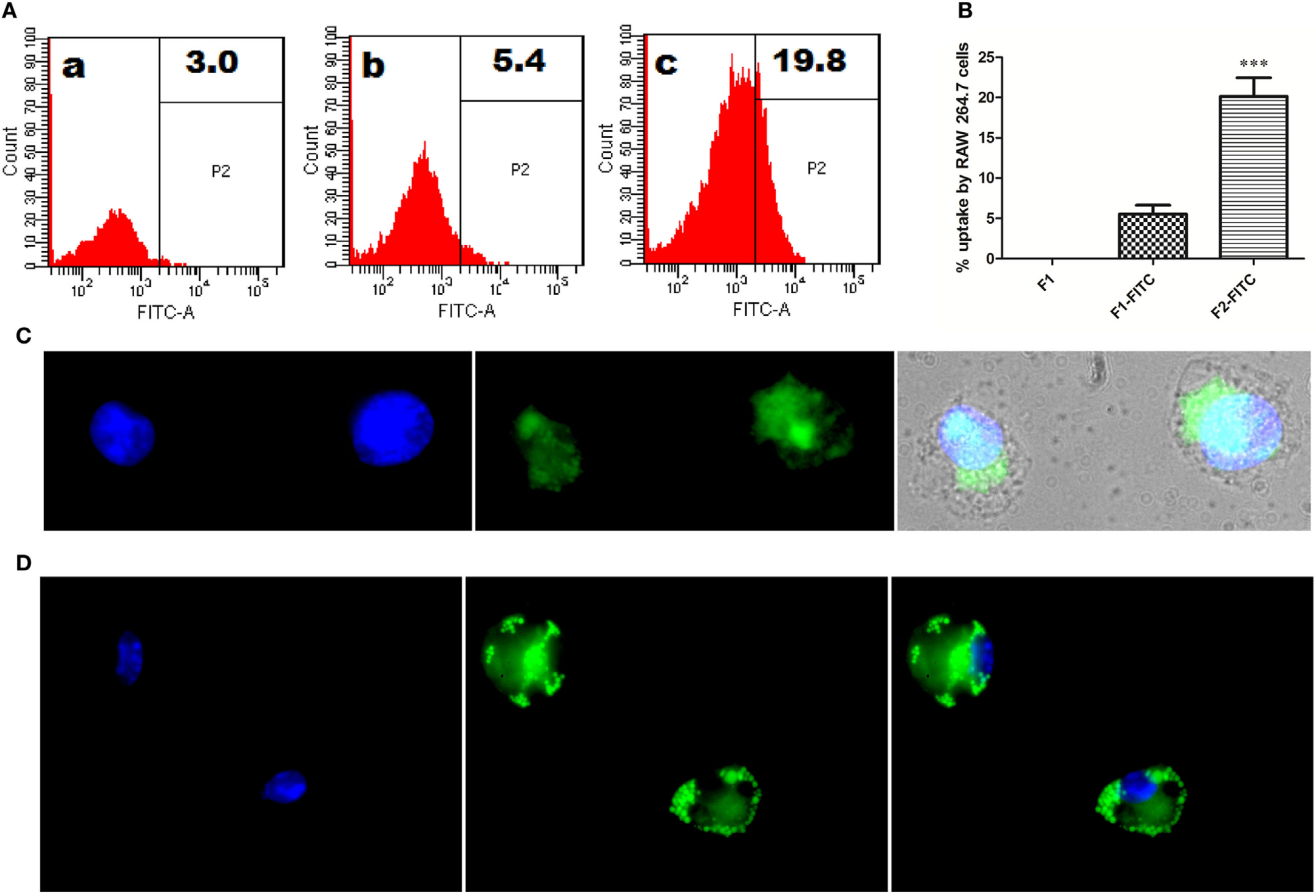
*In vitro* interaction between Ag85B aggregates and professional antigen presenting cells. **(A)** Uptake of aggregated Ag85B by RAW264.7 macrophages as revealed by FACS analysis. Representative gated histograms depicting cumulative uptake of (a) native Ag85B only (F1); (b) FITC labeled native Ag85B-IFA complex (F1-FITC); and (c) FITC labeled Ag85B aggregates (F2-FITC). **(B)** Bar diagram displaying percent uptake of aggregated Ag85B by RAW264.7 cells. Quantitative data corresponding to FACS mediated uptake analysis of amyloidal Ag85B and controls. Data represent the means ±SD from three independent experiments and normalized to background fluorescence recorded in unlabeled F1 sample. **(C)** Fluorescence microscopy depicting uptaken aggregated antigen by APCs. RAW264.7 macrophages were incubated for 6 h with aggregated Ag85B (F2) and then stained with ThT for amyloid labeling and subsequently imaged (nuclei stained blue, while ThT labeled antigen in green; 100× oil immersion lens was used). **(D)** Immuno-localization of aggregated Ag85B inside RAW264.7 macrophages. The protein aggregate-mediated cytosolic delivery of Ag85B was probed with monoclonal primary antibodies against native Ag85B and its subsequent detection with Alexa Fluor 488 tagged secondary antibody (nuclei stained blue and antigen in green, 100× oil immersion lens).

To follow the fate of fibrillar Ag85B upon its interaction with antigen presenting cells, uptake of aggregated antigen by macrophages was assessed employing flow cytometry and fluorescence microscopy. The fluorescein isothiocyanate (FITC) labeled fibrillar Ag85B or native Ag85B was incubated with RAW264.7 cells for 6 h (37 °C, 5% CO_2_) to quantitate antigen uptake. As per flow cytometry based evidence, the magnitude of FITC labeled fibrillar Ag85B accumulated in the cells was several folds high, indicating its better uptake when compared with free form of soluble protein (Figure 3A). The percentage uptake of labeled native antigen (F1-FITC) and labeled fibrillar antigen (F2-FITC) was also determined and are presented as a bar diagram. Background fluorescence generated by unlabeled native antigen (F1) incubated cells was considered as a baseline and manually subtracted from the F1-FITC and F2-FITC, respectively, before plotting on the graph (Figure 3B).

Uptake of fibrillar Ag85B by RAW264.7 macrophages was also probed by exclusive labeling of amyloidal antigen with ThT followed by its detection employing fluorescence microscopy. As expected, the antigen uptake resulted in diffuse fluorescence distributed across the interior of the target cells (Figure 3C). The diffuse fluorescence pattern (usually a signature of phagocytosed particulate antigen) of the target cells establishes the evidence for cytosolic delivery and subsequent class 1 processing of the internalized fibrillar antigen in the macrophages.

Further, immunofluorescence microscopy was also used to follow the fate of fibrillar Ag85B upon its interaction with RAW264.7 cells. The presence of fibrillar antigen was probed using primary antibodies (monoclonal) against native Ag85B that were subsequently detected with Alexa Fluor 488 tagged secondary antibodies. The depicted corresponding immuno-localization data (Figure 3D) clearly affirms the presence of delivered antigen across the cell interior. The observation establishes potential of fibril cargo to deliver the precursor antigen (native Ag85B) into cytosol of the target cells.

### Immunization with Fibrillar Ag85B Elicited Predominantly Th1 Type Cytokines

**Figure 4:**
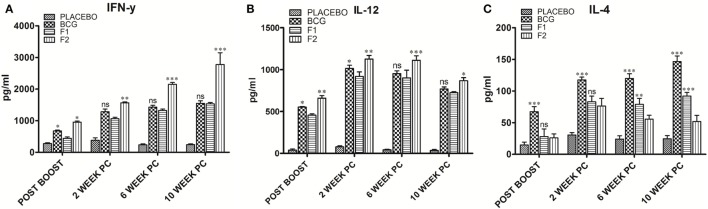
Ag85B aggregate mediated upregulation of Th1 cytokines in the immunized animals. Assessment of Ag85B aggregate-mediated Th1/Th2 bias, as ascertained by determining cytokine response (employing sandwich ELISA) in different immunized groups at various time points; **(A)** IFN-γ, **(B)** IL-12, and **(C)** IL-4. To induce *ex vivo* cytokine expression, splenocytes from groups of animals immunized either with Rv1886-IFA (F1) or aggregated Ag85B (F2) were stimulated with corresponding set of matching antigens (5 µg/ml). Lymphocytes isolated from Bacillus Calmette–Guerin (BCG) group were stimulated with BCG whole cell antigen (10 µg/ml) while cells from placebo immunized group were primed with whole cell antigen of *Mtb* H_37_Rv (10 µg/ml). The data were analyzed by employing Two-way ANOVA following by Bonferroni’s multiple comparison test and are shown as the means (±SD) of three independent determinants where; *p* < 0.05(*), *p* < 0.01(**), and *p* < 0.001(***) were considered significant.

The levels of signature cytokines of both Th1 (IFN-γ and IL-12) and Th2 (IL-4) arms of adaptive immunity were analyzed at various time intervals in splenocyte culture supernatant belonging to various immunized groups. Augmentation of Th1 type of cytokines is supposedly an essential prerequisite to establish conducive conditions that help in effective clearance of *Mtb* and other intracellular pathogens. The Th1 cytokine profile (Figures 4A,B), as assessed in culture supernatant by sandwich ELISA, clearly shows that the immunization with fibrillar Ag85B (F2) markedly raised the levels of Th1 cytokines as compared to native form of antigen with IFA (F1) or BCG (*p < 0.05). On the contrary, levels of Th2 cytokines decreased (low expression) in mice belonging to either F1 or F2 groups, in comparison to BCG immunized group (Figure 4C, ***p < 0.001). Further, the differences in IL-4 level were not significantly different initially (upto 2 weeks) between F1 and F2 immunized groups, but 6 weeks onward, the difference became more prominent (Figure 4C, **p < 0.01). The observed polarization suggests induction of Th1 favoring cytokine paradigm upon immunization of animals with amyloid form of antigen. There was no significant difference between Th1 cytokine production in BCG immunized group of animals and those immunized with native Ag85B-IFA (F1) at various PC time points, with only exception being the 2-week PC, where IL-12 was slightly higher in BCG immunized animals (Figure 4B, *p < 0.05). However, the post booster levels of two cytokines, IFN-γ and IL-12, moderately elevated in BCG group (Figures 4A,B, *p < 0.05). No significant alteration in expression of either Th1 or Th2 cytokines was found in placebo immunized group neither at post booster nor at post infection time points when compared to other immunized groups. Small elevation in IL-4 levels in placebo immunized animals observed PC time points may be attributed to non-specific activation of the immune system due to chronic TB infection (Figure 4C) (*p* > 0.05). Alternatively, it can also be considered as specific ability or strategic plan of *Mtb* to befool host immune system to induce pro pathogen type 2 cytokines.

### Ag85B Aggregate Based Immunization Schedule Ensues in Systemic Proliferation of T Lymphocytes and Production of Mostly IgG2a Subtype Antibodies

**Figure 5:**
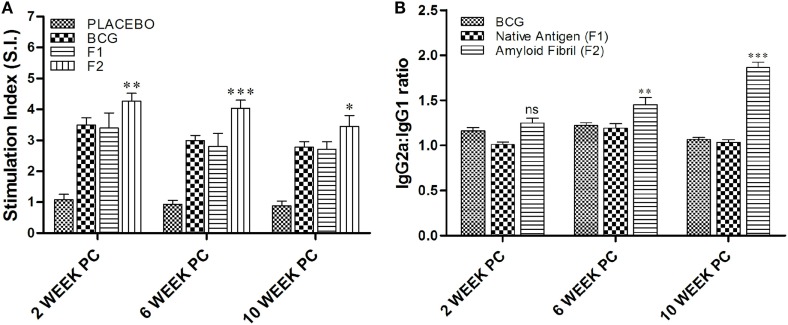
Evaluation of splenocyte proliferation potential and antibody isotype switching. **(A)** *In vitro* splenocyte proliferation response as deduced by MTT assay. At various time points post challenge, lymphocyte proliferation assay was performed to measure antigen-specific T lymphocyte proliferation. Lymphocytes isolated from various experimental groups were stimulated with corresponding set of matching antigens for 72 h. MTT solution (100 μl/well) was added in last 4 h (of incubation), and the proliferation induced was measured as described in Section “Materials and Methods.” The results presented are means (±SEM) of Stimulation Index (SI) from three independent experiments. The data were analyzed by two-way ANOVA following by Bonferroni’s multiple comparison test where; *p* < 0.05(*), *p* < 0.01(**), and *p* < 0.001(***) were considered significant. **(B)** Antibody isotype switching in response to immunization with aggregated Ag85B. The isotype switching upon immunization with Ag85B aggregate was analyzed by ascertaining IgG2a and IgG1 isotypes in the sera employing sandwich ELISA and presented as ratio of the ODs obtained for IgG2a and IgG1. The data were analyzed by employing two-way ANOVA followed by Bonferroni’s multiple comparison test and are shown as the means (±SEM) of three independent determinants where; *p* < 0.05(*), *p* < 0.01(**), and *p* < 0.001(***) were considered significant.

MTT [3-(4,5-dimethylthiazol-2-yl)-2,5-diphenyltetrazolium bromide, a tetrazole]-based T cell proliferation assay was employed to determine T cell proliferative potential of fibrillar Ag85B. As evident in Figure 5A, a strong antigen-specific proliferative response was seen in splenocytes belonging to animals immunized with fibrillar Ag85B (F2) consistently at 2 (**p < 0.01), 6 (***p < 0.001), and 10 (*p < 0.05) weeks post challenge compared to those observed for BCG immunized animals. No significant difference was, however, observed in proliferation of splenocytes belonging to BCG and F1 immunized group of animals (*p* > 0.05). Immunization with F2 resulted in significantly high proliferative response in splenocytes when compared to BCG or F1 immunized animals at various time points PC (*p < 0.05). Only baseline level of proliferation was evident in splenocytes of PB immunized animals (placebo control group) when compared to other experimental groups (***p < 0.001).

Next, we investigated antibody isotype class switching in the mice immunized with various forms of Ag85B antigen. The results of the study, as shown in Figure 5B, clearly depict that the administration of amyloid based antigen resulted in significantly higher levels of IgG2a in immunized animals (**p < 0.01). The IgG2a/IgG1 isotype ratio in F2 immunized animals was not found to be significantly different from BCG and F1 immunized groups at 2 weeks PC; however, the ratio was markedly higher in F2 immunized group at 6 and 10 weeks PC when compared to both BCG and F1 groups with maximal elevation observed at 10 weeks post challenge (***p < 0.001). In BCG immunized animals, isotype ratio remained 1.2 at 2 and 6 weeks PC, while at 10 weeks PC it was slightly dropped to around 1, suggesting equal abundance of two isotypes that eventually led to the Th2-dominant environment.

### Immunization with Fibrillar Ag85B Evokes Polyfunctional T Cells

**Figure 6:**
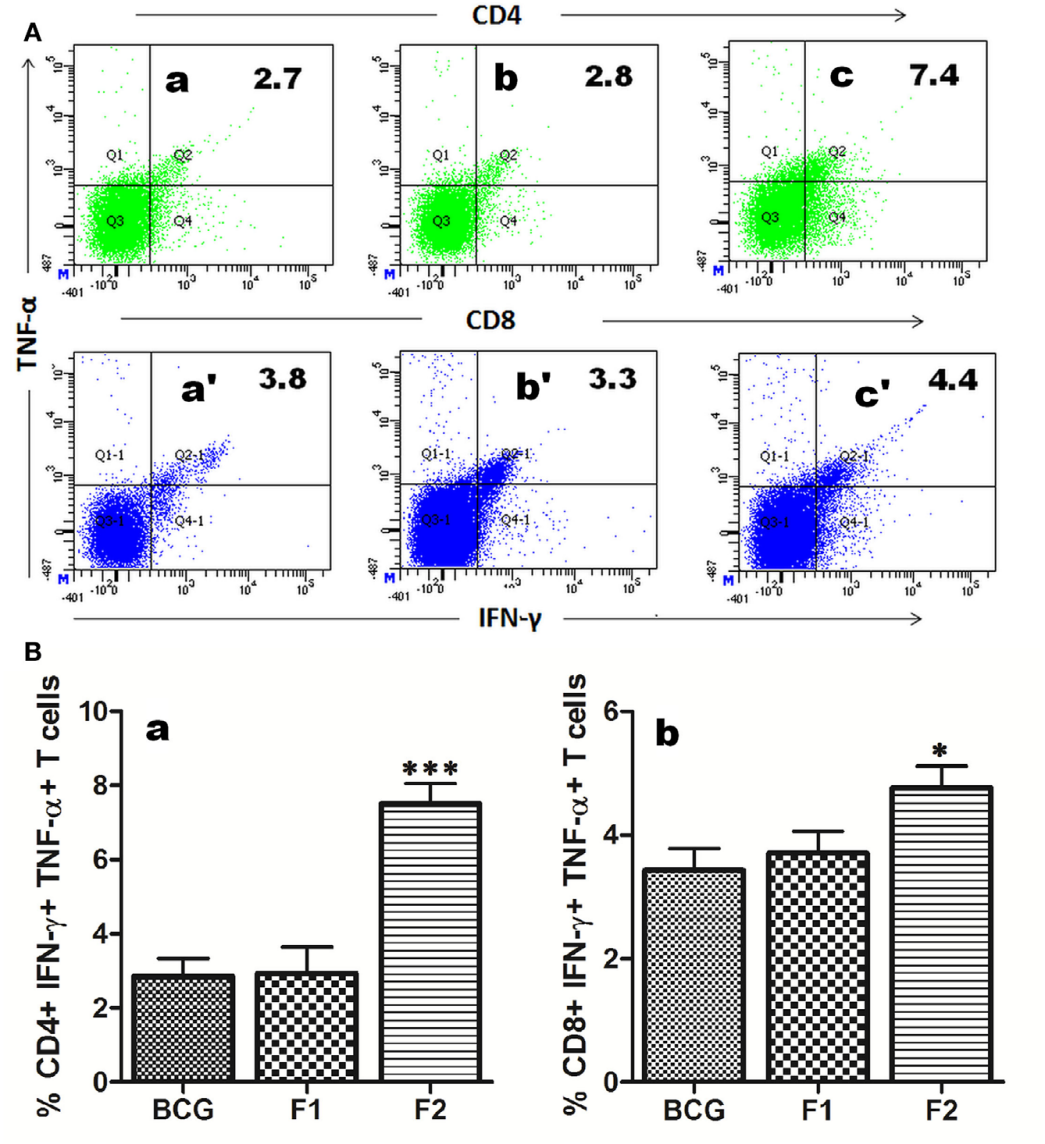
Immunization with aggregated Ag85B results in expansion of polyfunctional T cells in immunized mice. T cells isolated from mice immunized with Bacillus Calmette–Guerin (BCG), Ag85B-IFA (F1), and aggregated Ag85B (F2) were stimulated *ex vivo* with corresponding antigens for 24 h and stained for intracellular IFN-γ and TNF-α. **(A)** Representative FACS dot plots denoting cumulative frequencies of CD4^+^ and CD8^+^ polyfunctional T cells. Cells co-expressing IFN-γ and TNF-α on immunization with BCG (a and a′), native Ag85B-IFA (F1) (b and b′), and aggregated Ag85B (F2) (c and c′). **(B)** Bar graphs depicting percent population of IFN-γ, TNF-α co-expressing CD4^+^ (a), and CD8^+^ (b) polyfunctional T cells. The cells were isolated from splenocytes of various immunized groups of mice at 1 week post booster. Dot plots are representative of at least three animals for each group. The data were analyzed by employing one-way ANOVA followed by Bonferroni’s multiple comparison test and shown as means (±SD) of three independent determinants, where *p* < 0.05(*), *p* < 0.01(**) and *p* < 0.001(***) were considered significant.

The multifunctional or polyfunctional T cells are reported to be crucial in providing protection against infection involving *Mtb* and other intracellular pathogens and emerged as an indispensable correlate of the protective immunity (12, 19, 21–23). Both CD4^+^ and CD8^+^ T cells generated in various immunized groups were assessed for their potential to simultaneous expression of multiple effector cytokines (IFN-γ and TNF-α). Interestingly, administration of fibrillar Ag85B resulted in proliferation of significantly high numbers of both CD4^+^ (***p < 0.05) and CD8^+^ (*p < 0.05) T cells expressing IFN-γ and TNF-α concomitantly, a hallmark of qualitatively superior T cells (23), when compared to BCG and Ag85B-IFA (F1) (Figure 6).

### Co-Stimulatory Potential of APCs was Induced upon Immunization with Fibrillar Ag85B

**Figure 7:**
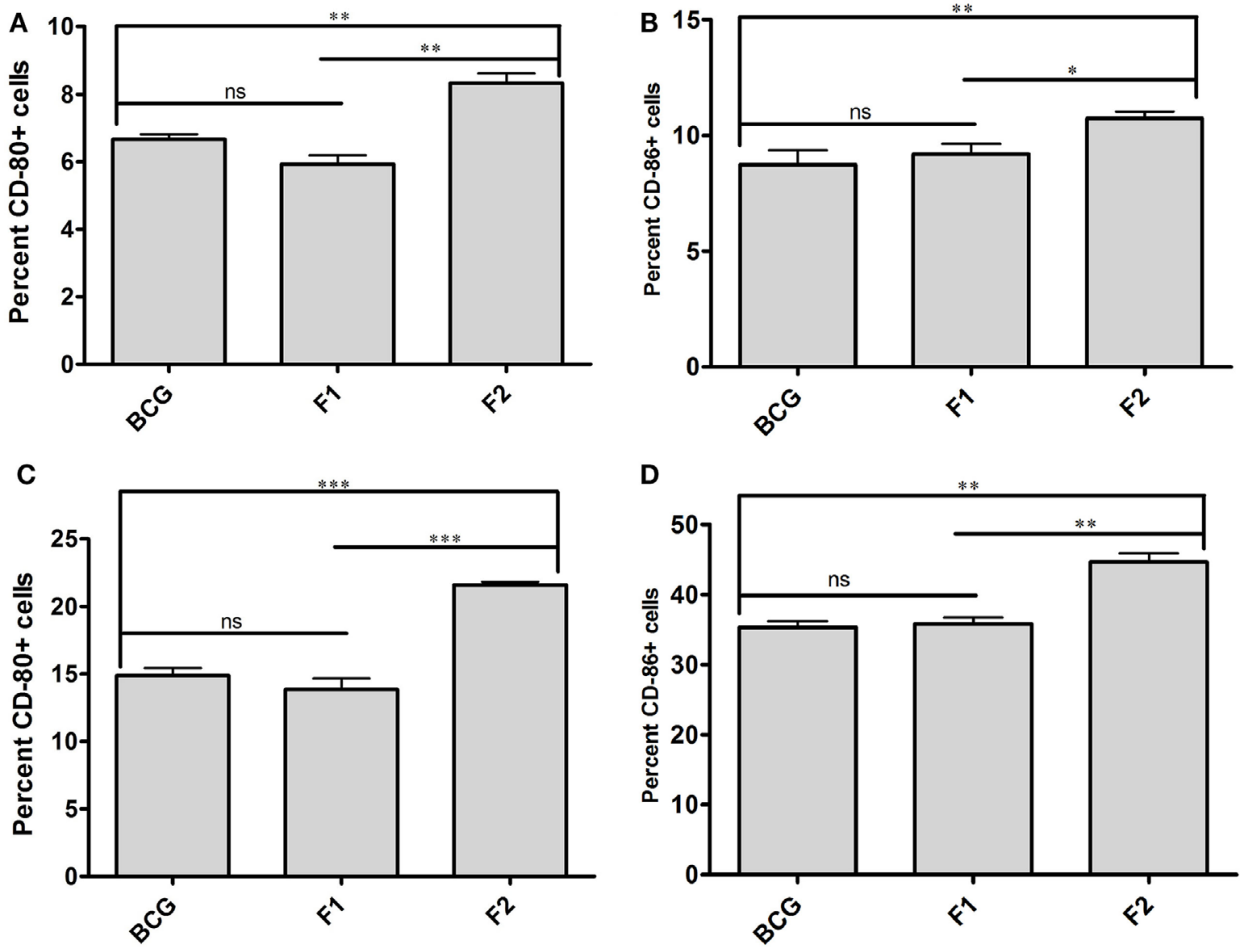
Aggregated Ag85B upregulates expression of co-stimulatory molecules on APCs. Expression level of signature co-stimulalotry molecules CD80 (B7.1) and CD86 (B7.2) on isolated CD11c^+^ APCs belonging to various experimental groups, namely; Bacillus Calmette–Guerin (BCG), Ag85B-IFA (F1), and aggregated Ag85B (F2) were determined through flow cytometry at various time points. The data correspond to 6 **(A,B)**, and 10 **(C,D)** weeks post challenge time points, respectively. The experiment was repeated thrice and data represent means (±SEM) from three independent experiments. Various immunized groups were compared to determine statistical significance of the data using One-way ANOVA following by Bonferroni’s multiple comparison test analysis with *p* < 0.05(*) as minimum level of significance.

Surface staining of APCs (CD11c^+^) isolated from various immunized groups of animals was performed to determine expression of prime co-stimulatory markers (CD80/86) on cell surface. Administration of either BCG or native Ag85B-IFA (F1) was resulted in upregulation of co-stimulatory molecules in almost similar fashion at 6 and 10 weeks PC (Figures 7A–D, *p* > 0.05). Intriguingly, immunization with fibrillar Ag85B (F2) resulted in dramatic upregulation of both CD80 and CD86 molecules on target cells when compared to the groups immunized with either BCG or native antigen (F1) (Figures 7A–D, *p < 0.05). Flow cytometry data pertaining to co-stimulation potential adjunct the evidence that the fabricated fibrillar vaccine delivery system of Ag85B was capable of inducing significantly higher expression of co-stimulatory molecules (CD80/86), when compared to the native antigen (F1).

### Improved T Cell Memory Response was Evident following Immunization with Fibrillar Ag85B

**Figure 8:**
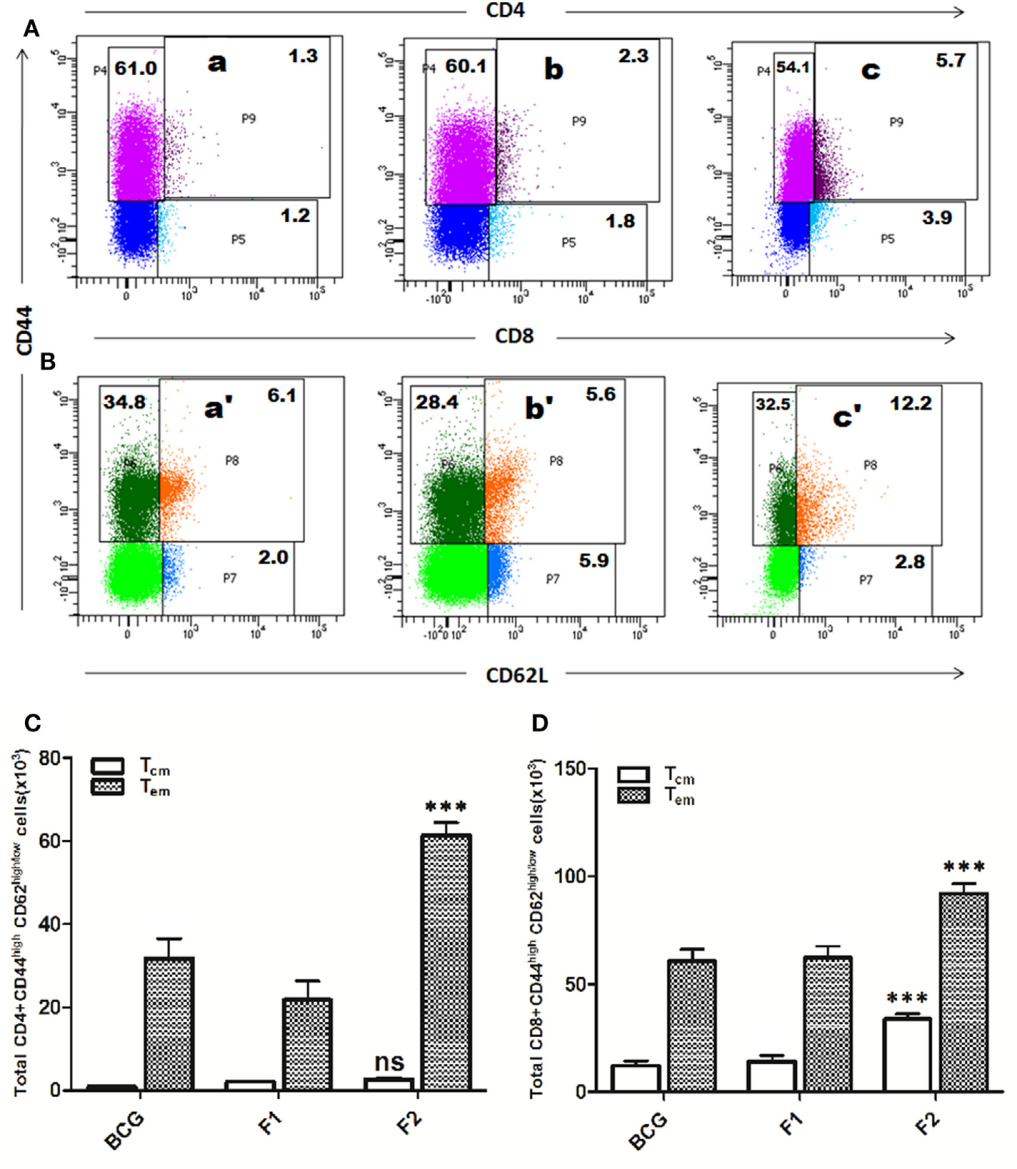
Immunization schedule involving Ag85B aggregates evokes antigen-specific T cell memory (effector/central) response in the host. The splenocytes were isolated from various immunized groups at 1 week post booster and analyzed for the presence of CD44^high^, CD62L^low/high^ CD4^+^, and CD8^+^ T cells. The representative FACS dot plots of gated **(A)** CD4^+^ T cells, and **(B)** CD8^+^ T cells belonging to various immunized groups are denoted in the upper panel. Various experimental groups included in the study were: Bacillus Calmette–Guerin (BCG) (a and a′), Ag85B-IFA or F1 (b and b′), and amyloidal aggregate form of Ag85B or F2 (c and c′). Graphs in the lower panel **(C,D)** depict quantitave data of analyzed memory markers belonging to corresponding CD4^+^ and CD8^+^ T cells. Bar graphs indicate total number of CD4^+^ CD44^high^, CD62L^low/high^ **(C)**, and CD8^+^ CD44^high^, CD62L^low/high^ **(D)** cells, respectively, in various groups. The data were analyzed by employing two-way ANOVA followed by Bonferroni’s multiple comparison test and are shown as means (±SD) of three independent experiments. **(C)** Aggregated Ag85B (F2) Vs BCG: *p* < 0.001 CD4^+^ T_effector memory_; *p* = ns CD4^+^ T_central memory_; Ag85B-IFA (F1) Vs BCG: *p* < 0.01 CD4^+^ T_effector memory_; *p* = ns CD4^+^ T_central memory_, aggregated Ag85B (F2) Vs Ag85B-IFA (F1): *p* < 0.001 CD4^+^ T_effector memory_; *p* = ns CD4^+^ T_central memory_. **(D)** Aggregated Ag85B (F2) Vs BCG: *p* < 0.001 CD8^+^ T_effector memory_, CD8^+^ T_central memory_; Ag85B-IFA (F1) Vs BCG: *p* = ns CD8^+^ T_effector memory_; CD8^+^ T_central memory_, aggregated Ag85B (F2) Vs Ag85B-IFA (F1): *p* < 0.001 CD8^+^ T_effector memory_; CD8^+^ T_central memory_.

**Figure 9:**
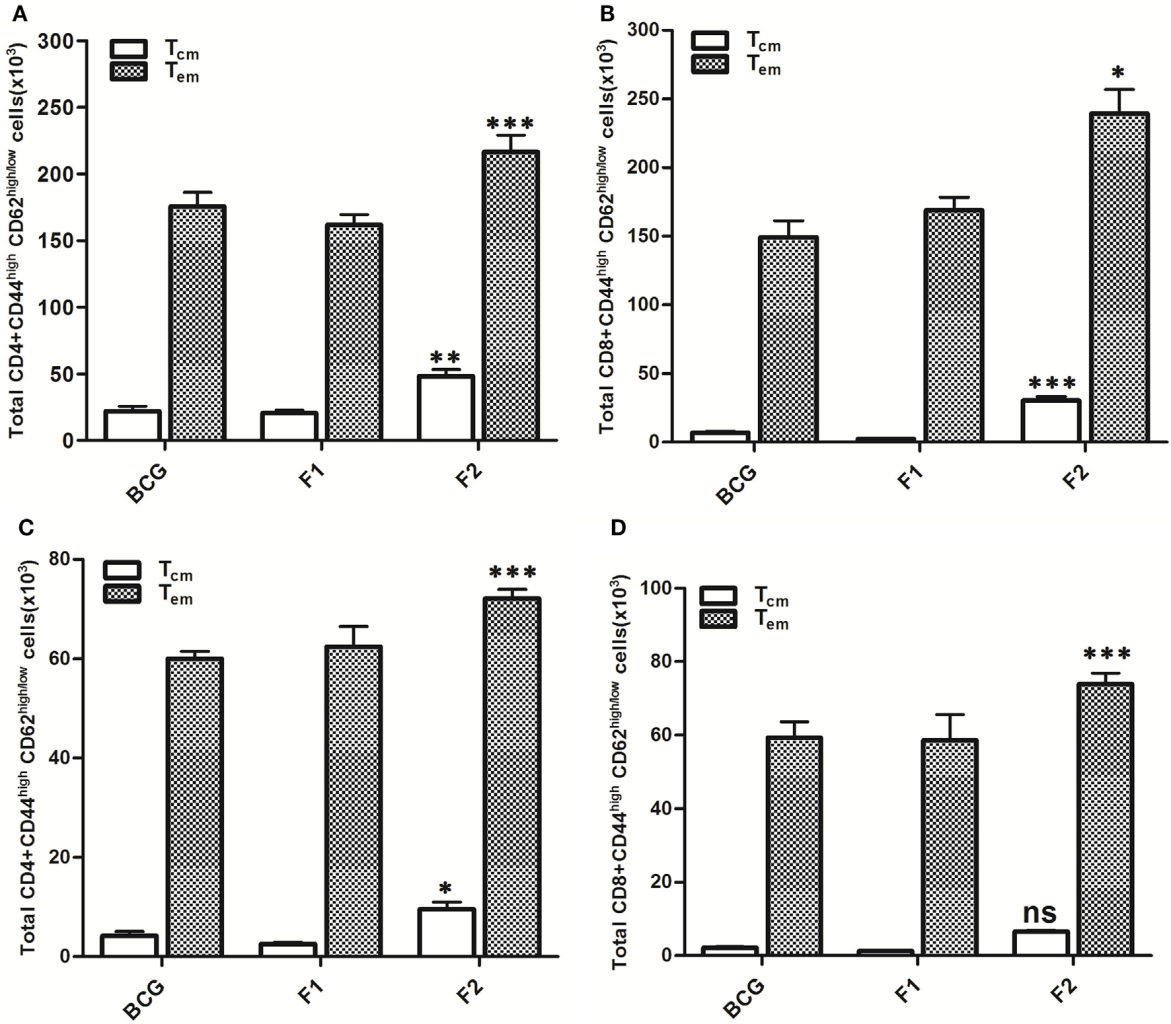
Aggregated Ag85B induces T lymphocytes with upregulated memory (effector/central) markers upto 10 weeks post challenge. After *Mtb* aerosol challenge, the splenocytes from various immunized groups were isolated and analyzed for the presence of CD44^high^, CD62L^low/high^ CD4^+^, and CD8^+^ T memory cells. Graphs in figure depict the quantitave data of FACS analyzed memory markers belonging to the corresponding CD4^+^ and CD8^+^ T cells. Bar graphs in upper panel indicate the total number of CD4^+^CD44^high^, CD62L^low/high^ **(A)**, and CD8^+^CD44^high^, CD62L^low/high^ **(B)** T cells, respectively, in various groups at 6 weeks post challenge and the bar graphs in lower panel **(C,D)** reflect the quantum of same memory cells at 10 weeks post challenge time point. The data were analyzed by employing two-way ANOVA followed by Bonferroni’s multiple comparison test and are shown as the means (±SD) of three independent experiments. CD4^+^ T cells 6 weeks **(A)** aggregated Ag85B (F2) Vs Bacillus Calmette–Guerin (BCG): *p* < 0.001 CD4^+^ T_effector memory_; *p* < 0.01 CD4^+^ T_central memory_ Ag85B-IFA (F1) Vs BCG: *p* = ns CD4^+^ T_effector memory_; CD4^+^ T_central memory_, aggregated Ag85B (F2) Vs Ag85B-IFA (F1): *p* < 0.001 CD4^+^ T_effector memory_; *p* < 0.01 CD4^+^ T_central memory_. CD8^+^ T cells 6 weeks **(B)** aggregated Ag85B (F2) Vs BCG: *p* < 0.05 CD8^+^ T_effector memory_; *p* < 0.001 CD8^+^ T_central memory_; Ag85B-IFA (F1) Vs BCG: *p* = ns CD8^+^ T_effector memory_; CD8^+^ T_central memory_, aggregated Ag85B (F2) Vs Ag85B-IFA (F1): *p* < 0.01 CD8^+^ T_effector memory_; *p* < 0.001 CD8^+^ T_central memory_. CD4^+^ T cells 10 weeks **(C)** aggregated Ag85B (F2) Vs BCG: *p* < 0.001 CD4^+^ T_effector memory_; *p* < 0.05 CD4^+^ T_central memory_; native Ag85B-IFA (F1) Vs BCG: *p* = ns CD4^+^ T_effector memory_; CD4^+^ T_central memory_, aggregated Ag85B (F2) Vs Ag85B-IFA (F1): *p* < 0.001 CD4^+^ T_effector memory_; *p* < 0.01 CD4^+^ T_central memory_. CD8^+^ T cells 10 weeks **(D)** aggregated Ag85B (F2) Vs BCG: *p* < 0.001 CD8^+^ T_effector memory_; *p* = ns CD8^+^ T_central memory_; Ag85B-IFA (F1) Vs BCG: *p* = ns CD8^+^ T_effector memory_; CD8^+^ T_central memory_, aggregated Ag85B (F2) Vs Ag85B-IFA (F1): *p* < 0.001 CD8^+^ T_effector memory_; *p* = ns CD8^+^ T_central memory_.

Lymphocytes isolated from various groups of immunized animals, at post booster as well as post *Mtb* aerosol challenge time points were analyzed for the presence of established cell surface memory markers (CD44/CD62L) employing flow cytometry. At 1 week post booster, both CD4^+^ and CD8^+^ T effector memory cells (CD44^high^CD62^low^) were found to be up-regulated significantly in the animals immunized with fibrillar Ag85B (F2). The T cells bearing central memory phenotype (CD44^high^CD62^high^) were also induced early in the CD8^+^ (***p < 0.001) as well as CD4^+^ (*p* > 0.05) T lymphocytes subsets belonging to Ag85B aggregate immunized animals (Figure 8). No significant expansion of memory T cells was seen either in BCG or native Ag85B-IFA (F1) immunized animals at any post booster time point. Further, when compared with BCG or Ag85B-IFA (F1) groups, the animals immunized with fibrillar Ag85B (F2) displayed higher magnitude of both CD4^+^ and CD8^+^ T cells with effector memory phenotype at 6 and 10 weeks post *Mtb* challenge (Figure 9, ***p < 0.001). T cells with central memory phenotype were also elevated in animals immunized with fibrillar Ag85B, when compared to those administered with BCG or native Ag85B-IFA (Figure 9, *p < 0.05) at 6 and 10 weeks PC. Although there was some downregulation in CD8^+^ T_cm_ level at 10 weeks PC (Figure 9D, *p* > 0.05), the quantum of CD8^+^ T_cm_ cells in Ag85B fibril immunized animals was still better at par with other immunized groups at 10 weeks post challenge.

### Immunization with Fibrillar Ag85B Confers Protection against Chronic *Mtb* Infection

**Figure 10:**
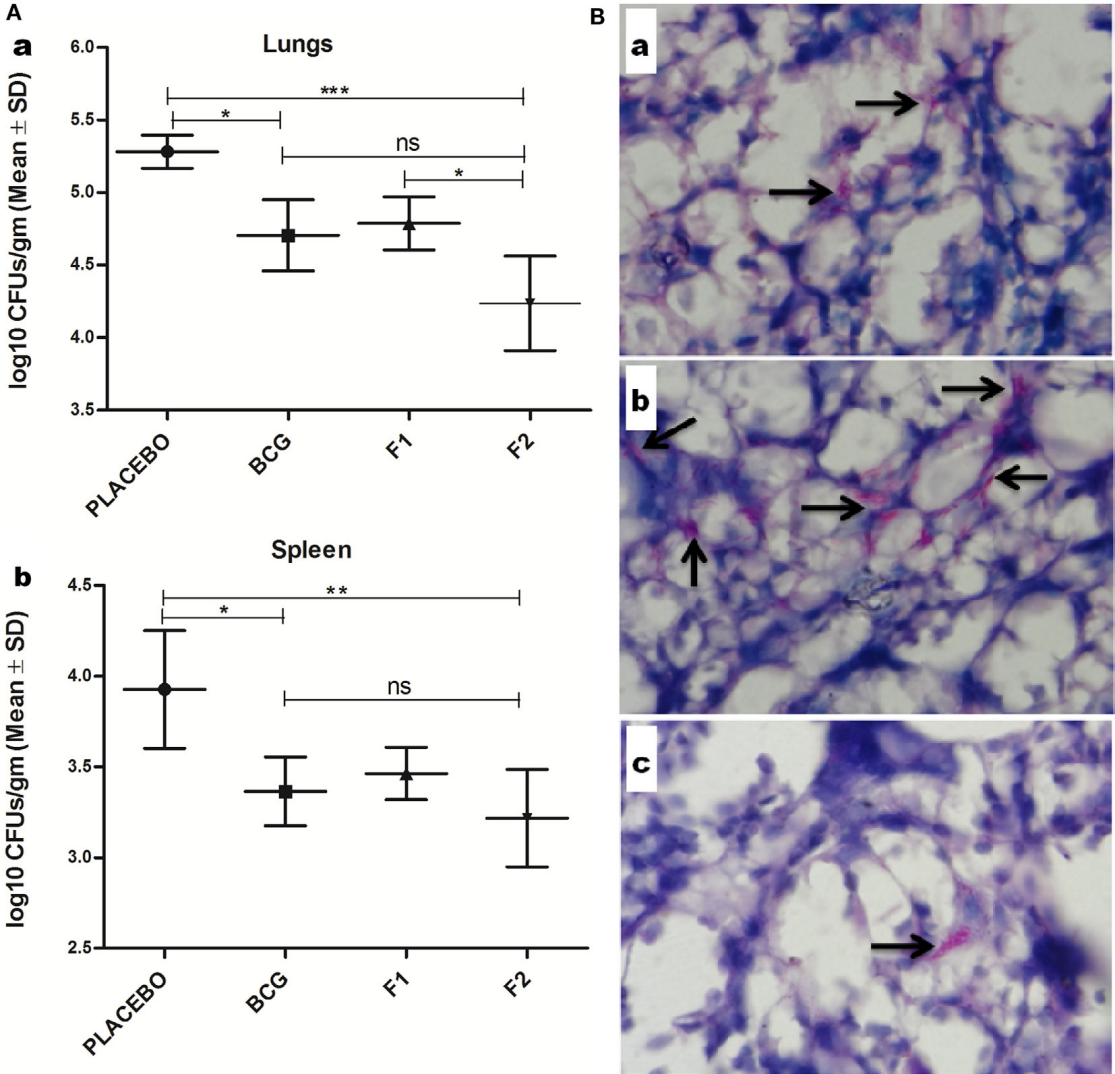
Evaluation of *Mtb* abundance in immunized mice at 10 weeks post infection. **(A)** Aggregated Ag85B suppresses *Mtb* bacillary load in the lungs and spleen of the immunized mice. Residual mycobacterial burden [colony-forming unit (CFUs)] in the lungs (a), and spleen (b) of the immunized animals was assessed at 10 weeks post infection. The colonies obtained were enumerated and expressed as log_10_ CFUs/g of tissue. The data were analyzed by employing one-way ANOVA following by Bonferroni’s multiple comparison test where; *p* < 0.05(*), *p* < 0.01(**), and *p* < 0.001(***) were considered significant. The log_10_ CFU data are expressed as means (±SD) of four animals in each group. **(B)** Histopathological analysis showing reduced residual mycobacterial burden in lungs of animals immunized with aggregated Ag85B. Representative histopathological photomicrographs from various immunized groups; (a) BCG, (b) Ag85B-IFA (F1), and (c) aggregated form of Ag85B(F2). Arrows pointing out relative occurrence of acid-fast bacilli positive bacilli (*Mtb*) in various immunized and infected groups at 10 weeks post aerosol challenge (Original magnification—1000×).

Prophylactic study was conducted to assess the prophylactic prospective of in-house formulated amyloid vaccine against low dose *Mtb* aerosol induced chronic TB. Immunized animals (belonging to various experimental groups) were euthanized (*n* = 4/group) at 10 weeks post *Mtb* challenge to enumerate residual bacterial load in their lungs and spleen. The bacterial burden was expressed as log_10_ CFUs/g (means ±SD of four animals from each group) (Figure 10A). Comparison of residual bacillary load in vital organs of various immunized groups was suggestive of striking prophylactic potential of fibrillar Ag85B. Immunization with fibril formulated Ag85B (F2) was found to impart better protection than BCG in terms of CFU reduction in lungs (Mean log_10_ reduction = 1.045, Placebo Vs F2, ***p < 0.001) and spleen (Mean log_10_ reduction = 0.708, Placebo Vs F2, *p < 0.05); when compared to placebo (vehicle buffer immunized) group of animals (Figure 10Aa,b). Surprisingly, F2-immunized animals exhibited enhanced clearance of bacilli from their lungs when compared to the animals immunized with F1 (*p < 0.05), which establishes an evidence favoring generation of better localized immunity at the primary site of infection upon immunization with amyloid form of antigen. Further, the reductions in log_10_ values of mycobacterial load upon immunization either with F1 or BCG were almost equivalent, when compared to placebo immunized animals. The data further substantiate the claim that amyloid-based vaccine delivery system indeed delivers the native form of antigen in a sustainable fashion that manifest generation of persisted immunological memory as well as augmented protection against subsequent pathogen invasion.

### Histopathological Study Revealed Lower *Mtb* Abundance in Lungs of Ag85B Aggregate Immunized Animals

Histopathological analysis of lung tissue from various immunized and infected animals was performed to examine and to correlate the *in situ* bacillary load in the lungs. The results obtained were suggestive of relatively low AFB-positive burden in animals vaccinated with fibrillar form of Ag85B (F2) and were almost comparable with those of BCG immunized animals. In addition, tissue specimen from the lungs of the animals belonging to the group immunized with native Ag85B-IFA (F1) contained relatively more number of tubercle bacilli, when compared to that presents either in F2 or BCG immunized animals. Collectively, the results of histopathology and AFB staining study further support the improved prophylactic efficacy of in-house fabricated fibrillar Ag85B antigen based vaccine in containing the mycobacterial infection (Figure 10B).

## Discussion

Since its inception, prophylactic intervention occupies center stage in eradication of various infectious diseases that inflict human race. For example, *Mycobacterium tuberculosis*, a pathogen that poses serious challenge to human health across the world, had been successfully controlled by BCG, a very assuring and globally acclaimed prophylactic vaccine till recent past. Ironically, the variable and abridged efficacy of the BCG, necessitates the development of unswerving alternatives to regulate the spectrum of morbidity and mortality caused by *Mtb* (24, 25). Replacing BCG is highly challenging yet desirable as it will address issues such as; (a) safety concerns associated with administration of BCG in HIV-infected individuals; (b) failure of BCG to sustain long-term T cell memory, and most importantly; (c) to circumvent the variable efficacy of BCG in TB-endemic regions.

Holding a distinction among various immunoprophylactic strategies, subunit vaccine is a system of choice against *Mtb* because it is likely to remain unaffected by exposure to environmental mycobacteria (26). Besides intrinsic attributes of an antigen, the specific immunological outcome of a subunit vaccine in the host is generally regulated by cytokine milieu, persistence slow release of native form of antigen and its access to proteasome machinery or endo-lysosomal degradation pathway, etc.

Several previous studies suggested that particulate antigen delivery system may affect its cross-presentation upon its internalization either *via* phago-lysosomal or vacuolar pathway (27–31). Nevertheless, delivery of antigen in cytosol of APCs for subsequent co-presentation of generated peptide along with MHC 1 molecules has always been considered an uphill task. Keeping this fact into consideration, we attempted to develop a novel prophylactic strategy employing amyloidal Ag85B based antigen delivery system. The particulate nature of the amyloidal aggregate based Ag85B subunit vaccine fosters its phagocytosis by professional antigen presenting cells. It is tempting to believe that fibrillar Ag85B harboring antigen presenting cells may act as a secondary depot of the precursor native antigen that eventually help in its dissemination to various lymphoid organs of the body.

During viral invasion, the transfer of genetic material is executed by membrane-membrane fusion of viral particle with that of host cell plasma membrane. Similarly, the release of secretory proteins (outside the cell) is accomplished via involvement of signal peptides. In an analogy to such fusion events, one can speculate that the exposed hydrophobic residues of fibrillar Ag85B may render its fusion with plasma membrane and eventually facilitates its direct access to cytosol of the antigen presenting cell. Once internalized, the fibrillar depot may release monomeric form of Ag85B that will be marked by proteasome machinery followed by class 1 processing and presentation that eventually lead to induction of antigen-specific CTL generation.

Before assessing the vaccine potential of as-synthesized aggregated Ag85B (formed at 24 h incubation) in experimental animals, we prompted to determine its propensity to interact with macrophages, the professional antigen presenting cells. As deduced through flow cytometric analysis, the cellular uptake of aggregated antigen (F2-FITC) by RAW264.7 macrophages was substantially higher when compared to the native antigen (F1-FITC) (Figures 3A,B, ***p < 0.001). The fluorescence microscopic studies further substantiated the presence of internalized Ag85B in the cytosol of RAW264.7 macrophages (Figure 3C). The cytosolic delivery potential of aggregated Ag85B was probed by determining the presence of monomeric antigen inside the cell using monoclonal antibodies against native Ag85B (Figure 3D). The uptake and immuno-localization studies deciphering interaction of Ag85B aggregate and antigen presenting cells imply: (a) hydrophobic interaction of amyloidal aggregate with plasma membrane of target cells; (b) protein aggregate-mediated delivery of monomeric Ag85B to cytosol of target cells; (c) MHC class 1 processing of monomeric Ag85B present in cytosol; followed by co-presentation of generated peptide along with MHC-I molecules for subsequent elicitation of antigen-specific CTL response in the host. Earlier studies have also underlined the inherent ability of Ag85B^144–153^ (32), and some other secreted mycobacterial antigens to access cytosol of the APCs for their specific degradation by proteasome machinery that ensues in co-presentation of generated peptide along with MHC-I molecules (14, 32, 33).

Next, we evaluated the immunogenicity as well as vaccine potential of developed amyloidal formulation (F2) in an *in vivo* system. Interestingly, the Ag85B fibrillar aggregate based immunization led to significantly higher T cell proliferation as compared to both BCG and F1 (Ag85B-IFA) immunized groups at various time points post *Mtb* aerosol infection (Figure 5A, *p < 0.05). Besides proliferation of antigen-specific CD4^+^ T cells, the protein aggregate-based immunization strategy was also successful in induction of CD8^+^ T cells (CTLs) in the immunized mice. Mice immunized with aggregated Ag85B induced higher proliferation of CTLs at various time points post infection, when compared with other immunized groups (Figure S1 in Supplementary Material).

Ag85B fibrillar aggregates primarily induced Th1 cytokines in the host with feeble generation of Th2 cytokines (Figures 4A–C). In spite of being a T cell antigen, native Ag85B evoked significantly lower levels of Th1 cytokines as compared to Ag85B based fibrillar aggregate at various time points (Figures 4A,B, *p < 0.01). Moreover, the T cells induced upon immunization with Ag85B aggregates were found to be of superior quality in terms of their ability to express multiple cytokines (IFN-γ and TNF-α) simultaneously (Figure 6). The multifunctional T cells have been reported to possess an extraordinary ability to maintain long lasting immunity (>50 years) in small pox vaccinated population to keep in check the infection (23).

There was preponderance of IgG2a class of antibodies over IgG1 in the animals immunized with Ag85B aggregates (Figure 5B). It is well established that IFN-γ secreting Th1 cells favors the induction of IgG2a, while Th2 cells favors the preponderance of IgG1 sub class of antibodies (34). The data obtained from antibody isotyping experiments signify skewed augmentation in favor of Th1 response in the animals immunized with aggregated Ag85B antigen (F2), when compared to animals immunized either with Ag85B-IFA (F1) or BCG. Further, the immunization with Ag85B aggregates resulted in development of antibodies that simultaneously recognized both- fibril released as well as purified native form of Ag85B antigen (Figure 2C).

Besides priming of host immune system, induction of memory response is a hallmark of any ideal vaccine formulation (35). We observed that immunization schedule involving aggregated form of Ag85B upregulated expression of both central (CD44^high^CD62L^high^) as well as effector memory (CD44^high^CD62L^low^) phenotype on both CD4^+^ and CD8^+^ T cell subsets. Interestingly, both CD4^+^ and CD8^+^ T effector memory subsets were significantly upregulated in animals immunized with Ag85B aggregates as compared to other groups (Figures 8 and 9, ***p < 0.001). Elevated level of CD4^+^ T cells with central memory persisted in the animals immunized with aggregated Ag85B (F2) upto 10 weeks (Figure 9, *p < 0.05), while CD8^+^ T cells remained upregulated up to 6 weeks (Figure 8, *p < 0.05) post *Mtb* challenge. At post booster time point, CD8^+^ T lymphocytes with central memory phenotype were present in high numbers in the animals immunized with aggregated form of Ag85B (Figure 8D, ***p < 0.001). However, there was no significant change in CD4^+^ T cell population with central memory phenotype among various immunized groups (Figure 8C, *p* > 0.05). In general, central memory persisting after rapid clearance of acute infections is more effective in controlling intracellular pathogens (36). Compelling with the hypothesis, the observed high amplitude of CD8^+^ central memory T cells during acute phase of infection (as determined in the animals at 6 weeks post challenge) and persisting CD4^+^ central memory T cells upto 10 weeks post infection, had significant implication in suppression of *Mtb* infection. The observed regulated expansion of memory T cell subsets further approves the better vaccine potential of aggregated Ag85B in comparison to classical BCG.

Vaccine-induced CD4^+^ central memory T cells have been demonstrated to be of prime importance to offer long lasting protection against murine TB (37). On the other hand, the effector memory response has been reported to be induced by chronic infections (38), and is usually short lived. Induction of long lasting CD4^+^ central memory T cells in the immunized animals post infection, affirms the superior immunogenicity of in-house fabricated fibril based vaccine. The observed memory induction upon Ag85B aggregate (F2) administration can have direct correlation with prolonged antigen persistence. Further, aggregate/fibril mediated delivery of Ag85B in cytosol of the target cells may facilitate its processing *via* class 1 proteasome machinery to effect presentation of corresponding processed peptide by APCs and their subsequent recognition by both CD4^+^/CD8^+^ effector T cells (Figure S1 in Supplementary Material).

The protection studies suggest that the immunization with Ag85B aggregate (F2)-based candidate vaccine suppresses mycobacterial infection in lungs and spleen of *Mtb* challenged mice up to 10 weeks. Generally, the protective potential of most candidate vaccines against TB is assessed during acute phase (4 and 8 weeks PC); however, we considered it more appropriate to appraise prophylactic potential of the candidate vaccine during chronic stage of the infection that usually occurs at post 8 weeks challenge in mice. The residual bacterial burden as accounted by enumerating bacterial load (Figure 10A) and histopathology study (Figure 10B) explicitly suggested the fact that the immunization schedule involving aggregated Ag85B was able to impart protection comparable to that offered by BCG against *Mtb* aerosol induced chronic TB in experimental mice. Together, the data of the study suggest that the immunization with aggregated Ag85B resulted in the following:

(i) induction of antigen-specific polyfunctional T lymphocytes
(ii) improved CD4^+^ and CD8^+^ T cell memory responses
(iii) profound expansion of Th1 dominant effectors (IFN-γ, TNF-α, IL-12)
(iv) predominance of Th1 favored IgG2a subtype over Th2 favored IgG1 in the sera
(v) upregulation of co-stimulatory molecules (CD80/CD86) on the surface of APCs
(vi) lessened bacterial burdens in lungs and spleen of *Mtb* aerosol-challenged mice.

The observed immunological outcome is a proof of the concept that protein aggregate/amyloid-based vaccine delivery system provides a novel platform to deliver native precursor antigen in sustained manner that activates both humoral and cell mediated arms of immune system with desirable memory response. The Ag85B aggregate/fibril induced observed heightened immune responses could be ascribed to the following scenarios. First, amyloidal aggregate mediated prolonged antigen release could offer prime-boost effect on both CD4^+^ and CD8^+^ memory T cell generation. It has already been reported that antigen persistence during infection correlates with a stronger T cell response (39). The slow release of protein from aggregate protein depot may more closely approximate matching pattern of antigen release during an infection. Second, aggregate/fibril/amyloid, due to their specific recognition by TLRs and also their ability to access cytosol of APCs, may ensue in better adjuvant effect and eventually induce amplification of superior IFN-γ and TNF-α co-producing CD4^+^ and CD8^+^ T cells. Third, protein aggregate-based immunization results in balanced expansion of memory CD4^+^ and CD8^+^ T cells that elicit immediate effector functions in response to challenge with pathogen infection. The ensuing robust secondary prophylactic responses resulted in rapid clearance of the infection. In conclusion, the data of the study highlight that besides cytosolic delivery, the degree and magnitude of antigen release from the aggregate depot can manipulate outcome of overall immune response, including long-term maintenance of T cell memory and efficacy in pathogen recall.

Importantly, the novel protein aggregate-based vaccination strategy may have implications in immunizing human subjects with HIV background or those receiving immunosuppressive therapies. Further, studies with appropriate modalities are needed to delineate the underlying biophysical, molecular, and immunological events and pathways regulating the immunity offered by protein aggregate-based vaccine. Once worked out, it might prove to be a novel approach to develop better vaccination schemes against *Mtb* and other prevalent intracellular pathogens.

## Ethics Statement

All animal experiments were approved by the Institutional Animal Ethics Committees of Interdisciplinary Biotechnology Unit, AMU, Aligarh, India and National JALMA Institute for Leprosy and Other Mycobacterial Diseases, Agra, India. All animal experiments were performed according to the National Regulatory Guidelines issued by CPCSEA. Our approval ID was 332/CPCSEA, Ministry of Environment and Forest, Govt. of India.

## Author Contributions

FA wrote the first draft of the manuscript and performed all major experiments. MO, SZ, and FA conceived the idea and designed the experiments. MO and SZ provided technical guidance and oversight throughout the entire process. MO and FA analyzed the data and interpret the results. PG and FA performed animal challenge studies. FA and RP performed flow cytometry experiments and analysis during challenge studies. UG provided mice and facility (BSL-3) and supervised the challenge study. MO and FA together critically revised the manuscript with inputs from co-authors. All authors discussed the results and implications and commented on the manuscript at various stages.

## Conflict of Interest Statement

The authors declare that the research was conducted in the absence of any commercial or financial relationships that could be construed as a potential conflict of interest.

## Abbreviations

PB, phosphate buffer; *Mtb, Mycobacterium tuberculosis*; PC, post challenge; F1, native Ag85B with incomplete freund’s adjuvant; F2, fibrillar aggregates of Ag85B; ThT, thioflavin-T dye; CR, congo red dye; aggr, aggregates.
