# Supplementary material for: Evaluation of Aggregated Ag85B Antigen for Its Biophysical Properties, Immunogenicity, and Vaccination Potential in a Murine Model of Tuberculosis Infection
Source: Front Immunol. 2017 Nov 27;8:1608. doi: 10.3389/fimmu.2017.01608 (PMC5711810; doi:10.3389/fimmu.2017.01608)
Supplement: Supplementary file 1 [file data_sheet_1.pdf]

Supplementary Information

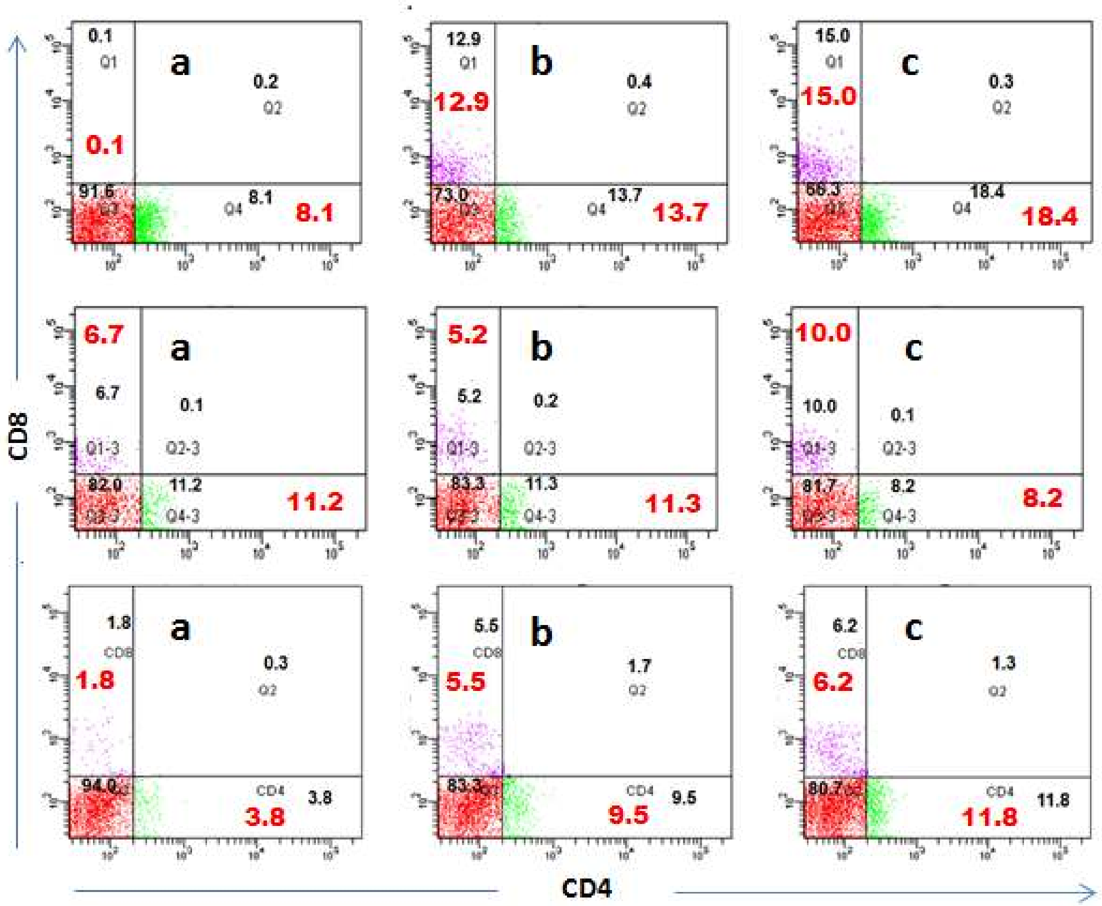

Fig.S1. Immunization with amyloidal Rv1886c (F2) resulted in better proliferation of CD8+ CTLs.

Shown in the figure are the representative FACS quadrant plots corresponding to percentage induction of both CD4+ & CD8+ T cells in the CD3 gated spenocytes of various immunized groups viz. BCG **(a),** Rv1886c-IFA or F1 **(b),** and amyloid form of Rv1886c or F2 **(c)**. The data in upper, middle and lower panels of the figure belongs to 2, 6 and 10 weeks post infection time points respectively.

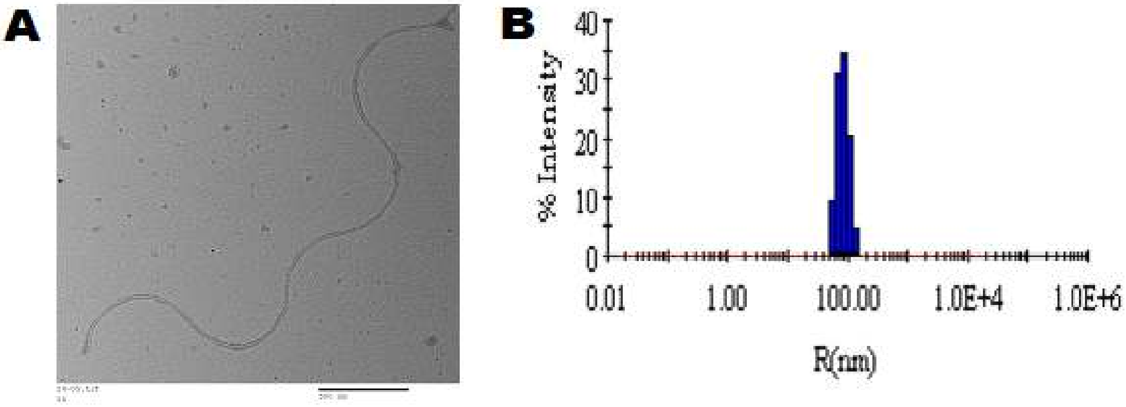

Fig.S2. Size analysis of as-synthesized Rv1886c amyloid (F2).

(A). Transmission Electron Micrography (TEM) image shows the agitation induced transformation of native Rv1886c into nano sized amyloidal structure. **(B)**. Dynamic Light Scattering (DLS) aided measurement of Rv1886c amyloid revealed average size of 77.46 nm and polydispersity index of 24.8%. Results are representative of at least three different experiments.
